# A Cyanobacterial Screening Platform for Rubisco Mutant Variants

**DOI:** 10.1021/acssynbio.5c00065

**Published:** 2025-07-07

**Authors:** Ute A. Hoffmann, Anna Z. Schuppe, Axel Knave, Emil Sporre, Hjalmar Brismar, Elias Englund, Per-Olof Syrén, Elton P. Hudson

**Affiliations:** † Department of Protein Science, School of Engineering Sciences in Chemistry, Biotechnology and Health, Science for Life Laboratory, 7655 KTH - Royal Institute of Technology, 106 91 Stockholm, Sweden; ‡ Department of Fibre and Polymer Technology, School of Engineering Sciences in Chemistry, Biotechnology and Health, Science for Life Laboratory, 7655 KTH - Royal Institute of Technology, 100 44 Stockholm, Sweden; § Department of Applied Physics, School of Engineering Sciences, Science for Life Laboratory, 7655 KTH - Royal Institute of Technology, 114 19 Stockholm, Sweden

**Keywords:** rubisco, high-throughput screening, cyanobacteria, *Synechocystis*, protein engineering, enzyme engineering

## Abstract

Rubisco is the main entry point of inorganic carbon into the biosphere and a central player in the global carbon system. The relatively low specific activity and tendency to accept O_2_ as a substrate have made Rubisco an attractive but challenging target for enzyme engineering. We have developed an enzyme engineering and screening platform for Rubisco using the model cyanobacterium *Synechocystis* sp. PCC 6803. Starting with the Form II Rubisco from *Gallionella,* we first show that the enzyme can replace the native Form I Rubisco in *Synechocystis* and that growth rates become sensitive to CO_2_ and O_2_ levels. We address the challenge of designing a zero-shot input library of the *Gallionella* Rubisco, without prior experimental knowledge, by coupling the phylogenetically guided model EV mutation with “*in silico* evolution”. This multisite mutagenesis library of *Synechocystis* (*n* = 16) was subjected to competitive growth in different gas feeds coupled to deep sequencing, in order to compare Rubisco variants. We identified an amino acid exchange that increased the thermostability of *Gallionella* Rubisco and conveyed resilience to otherwise detrimental amino acid exchanges. The platform is a first step toward high-throughput screening of Rubisco variants in *Synechocystis* and creating optimized enzyme variants to accelerate the Calvin–Benson–Bassham cycle in cyanobacteria and possibly chloroplasts.

## Graphical abstract

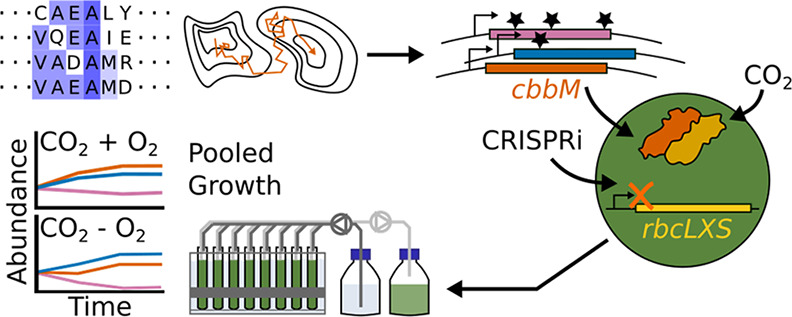

## Introduction

Most inorganic carbon enters the biosphere via fixation by Rubisco as part of the Calvin–Benson–Bassham cycle.^1,2^ Rubisco has been an important, though elusive target for enzyme engineering in efforts to improve growth, photosynthetic activity and productivity of photoautotrophs.^1–6^ Despite its central role in carbon metabolism, Rubisco exhibits a low turnover number (*k*_cat,C_) for a central metabolic enzyme.^7^ Further, in addition to the carboxylation of ribulose-1,5-bisphosphate (RuBP), Rubisco catalyzes the oxygenation of RuBP, a side reaction that leads to an energy-intensive salvage pathway (photorespiration^1^). The specific activity and substrate specificity of Rubisco may be connected, as evidenced from analyses of naturally occurring Rubiscos.^1,2^ For instance, a higher carboxylation rate *k*_cat,C_ often comes at the expense of a lower specificity *S* for CO_2_, particularly among the Form I Rubiscos of plants and algae, which have high sequence similarity.^1^ The trade-off is also present among Form II Rubiscos of bacteria, which are less complex than the hexadecameric Form I Rubisco. A systematic study found that while select Form II Rubiscos had significantly higher carboxylation rates than Form I Rubiscos, this was accompanied by a lower affinity for CO_2_ and lower *S*.^8^

Attempts to engineer the catalytic properties of Rubisco have been undertaken for several decades, often with the goal of enhancing the carboxylation rate of the enzyme.^1,2^ To facilitate Rubisco engineering, several bacterial selection platforms have been developed. These include facultative autotrophs such as or , as well as () strains which were engineered to be dependent on Rubisco activity for cell survival.^1^ Recently, the Form II Rubisco from () was subjected to barcoded deep mutational scanning (DMS) using a Rubisco-dependent strain. The genetic barcoding of Rubisco mutants enabled fitness of thousands of variants to be accessed via growth competition and deep sequencing.^9,10^ The study also highlighted the challenge of engineering Rubisco; while it was possible to alter enzyme selectivity by single amino acid exchanges, none of these exchanges resulted in a substantially more active enzyme, implying that several amino acid exchanges at once are needed to reach this goal.^10^ However, successful multisite engineering of proteins using random mutagenesis usually results in the vast majority of variants having a strong fitness decrease.^11–15^ One way to improve the chances of obtaining a functional protein during multisite mutagenesis is to use zero-shot prediction algorithms that incorporate prior knowledge on the protein, such as phylogenetic data.^16^ The computational method EVmutation^17,18^ was shown to perform well among zero-shot predictors in predicting the fitness of multisite protein variants across different protein phenotypes such as enzyme kinetic activity, ligand binding, and fluorescence.^15,16,19^ EVmutation models the evolutionary sequence covariation of a protein of interest by using multiple sequence alignments of homologues. This captures both the conservation of a residue at a given position as well as the pairwise covariation of amino acid positions.^17,18^ To address the challenge of picking residue positions to be included in a multisite zero-shot library, in this work we coupled predictions by EVmutation with “*in silico* evolution”, a variant of the Metropolis-Hastings algorithm.^20^ Similar methods were previously successfully employed to generate a high percentage of functional proteins with a high number of amino acid exchanges.^21–23^

To experimentally screen Rubisco variants, we use the model photosynthetic cyanobacterium *Synechocystis.* Cyanobacteria represent an emerging bioproduction system, and Rubisco has been implicated as partially limiting the synthesis rate of some chemicals by cyanobacteria.^24–26^ Furthermore, the autotrophic, and diurnal metabolism of cyanobacteria may provide metabolic states more similar to algae and plants than heterotrophic bacteria previously used for Rubisco screening. We considered the strong dependence of metabolite levels on *Synechocystis* growth condition^27–30^ an interesting set of constraints for developing new Rubiscos. To create a Rubisco screening strain, we expressed the Form II Rubisco from *Gallionella* (CbbM) in *Synechocystis,* and repressed the native Form I Rubisco using CRISPR interference (CRISPRi), so that cell growth became dependent on CbbM activity. Using EVmutation^17,18^ and *in silico* evolution,^20^ we identified four amino acid exchanges predicted to improve CbbM fitness, which we combined in various ways to generate 16 combinatorial variants of CbbM. We performed a pooled growth competition of the *Synechocystis* expressing these different variants in different gas feeds and tracked growth of each mutant clone using barcode sequencing. None of the predicted exchanges improved growth of *Synechocystis* compared to CbbM, again suggesting that enhancing Rubisco specific activity is a challenging protein engineering task. However, even within the 16 variants tested, we found epistatic relations. Two of the amino acid exchanges were able to rescue the detrimental effect of the other two. Biochemical characterization of CbbM mutants also showed that some had increased thermal stability. This screening platform is easily scalable to larger Rubisco libraries, and will thus enable high-information Rubisco engineering in the context of photosynthesis metabolism.

## Results and Discussion

### Efficient Repression of *Synechocystis* Rubisco, RbcLS, Using CRISPR Interference

The aim of this study was to engineer a *Synechocystis* host strain to be used as an in vivo screening tool where the activity of heterologously expressed Rubisco affects the host’s growth rate. We chose the Form II Rubisco from *Gallionella* (CbbM) as the heterologous Rubisco. Compared to Form I Rubiscos, Form II Rubiscos are usually more easily expressed heterologously due to their lower dependence on chaperones.^2,8^ Furthermore, the need to maintain protein–protein interactions, e.g. with Rubisco-specific assembly factors or the small subunit, might constrain the evolvability of Form I Rubisco large subunits compared to other forms.^6,31,32^ CbbM forms a dimer (L_2_) and has a significantly higher carboxylation rate (k_cat,C_ 22.2 ± 1.1 s^–1^) compared to plant Rubiscos, but a lower specificity (*S* 10.0 ± 0.1).^8^

As a first step, we depleted endogenous Rubisco activity using inducible CRISPRi with two single guide RNAs (sgRNAs) targeting the first 70 bp of the *rbcL* coding sequence (Figure 1A). A transcriptional repression of *rbcL* targets the whole *rbcLXS* operon, an operon that is essential for both photoautotrophic and heterotrophic growth.^33,34^ We used mass spectrometry to compare the depletion of RbcLS in strains harboring either the gene for dCas9 alone (*Syn*-sgRNA­(−)), or the complete CRISPRi construct including dCas9 and the *rbcLXS*-specific sgRNAs (*Syn*-sgRNA*_rbc_*) (Figure 1B and Supp. Figure S1A,B, Supp. Tables S1, S2). Only 1% of RbcL and 5% of RbcS were detected in *Syn*-sgRNA*_rbc_* compared to *Syn*-sgRNA­(−). We observed a similar trend for a strain containing the complete CRISPRi system and complemented with *Synechocystis* codon-optimized CbbM expressed from a plasmid (*Syn*-sgRNA_*rbc*_ *cbbM*(WT)) (2% of RbcL, 3% of RbcS, Figure 1B and Supp. Figure S1A–D, Supp. Tables S1–S4). The observed depletion matches our prior experiences of approximately 75% transcript repression using the CRISPRi system when targeting a variety of other genes, e.g. *glgC*,^35^ *gltA*,^36^ or *gyrA*.^37^

**Figure 1:**
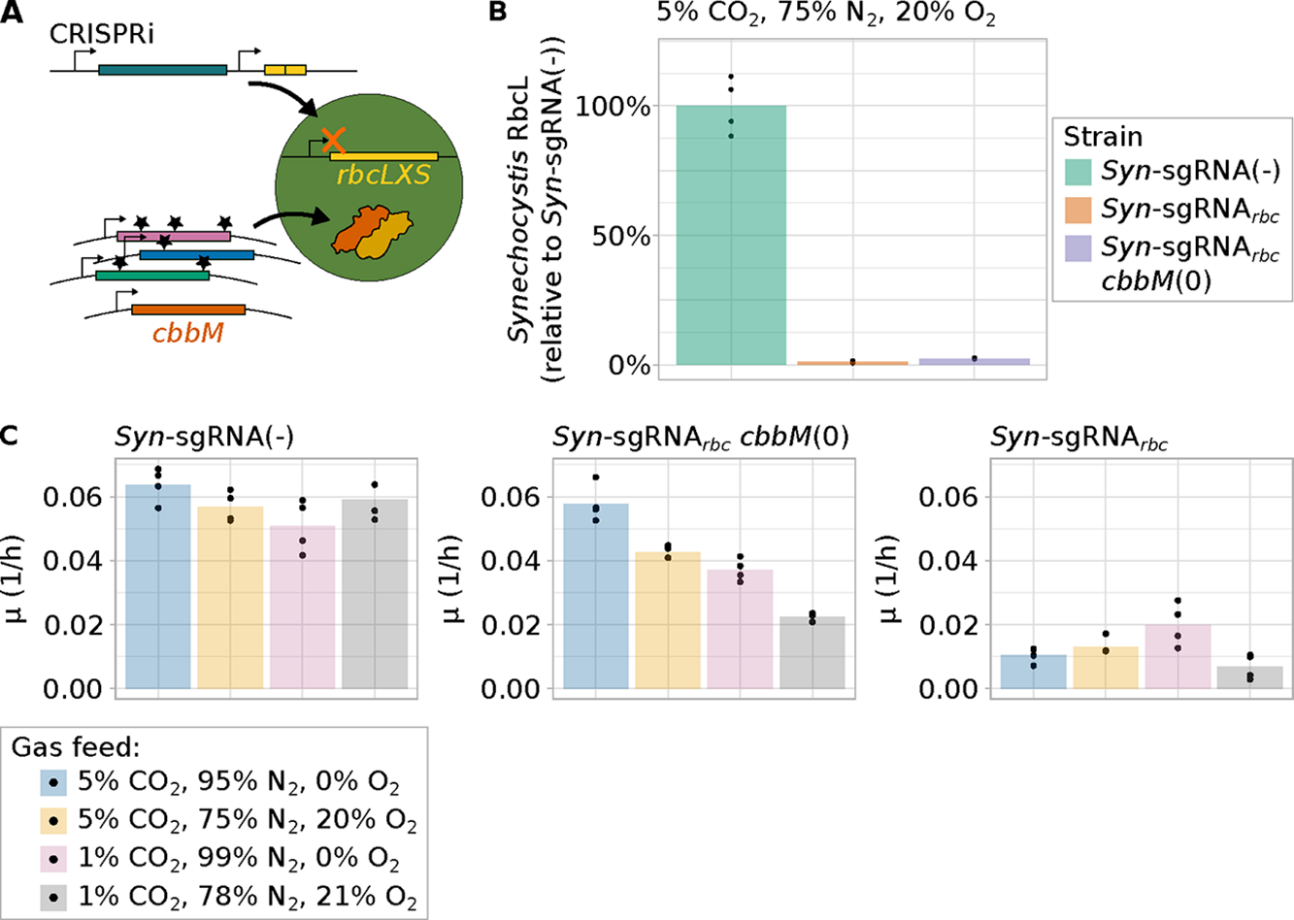
Characterization of the *Synechocystis* host strain used for Rubisco screening. (A) Schematic of method for screening of CbbM variants. CRISPR inhibition (CRISPRi) was used for transcriptional repression of the *rbcLXS* transcript encoding the endogenous Rubisco of *Synechocystis*. The gene *cbbM*, which encodes *Gallionella* Rubisco, CbbM, was introduced into the host strain under the control of a constitutive promoter. (B) Relative amount of *Synechocystis* Rubisco large subunit RbcL measured by mass spectrometry comparing *Syn*-sgRNA­(−), *Syn*-sgRNA*_rbc_* and *Syn*-sgRNA_*rbc*_ *cbbM*(WT) after approximately five generations of induction of the CRISPRi system using anhydrotetracycline (aTc). Signal intensity for RbcL is shown relative to the mean of the values measured for the replicates of *Syn*-sgRNA­(−). Bar plots represent the mean of four replicates, which are shown as single dots. For non-normalized data and data for the small subunit RbcS, see Supp. Figure S1. (C) Specific growth rates of *Syn*-sgRNA­(−), *Syn*-sgRNA*_rbc_*, and *Syn*-sgRNA_*rbc*_ *cbbM*(WT) calculated from batch cultivations in different CO_2_/O_2_ ratios as gas feed, after induction of the CRISPRi system using anhydrotetracycline (aTc, *n* = 4, *n* = 3 for *Syn*-sgRNA_*rbc*_ *cbbM*(WT) at 1% CO_2_, 78% N_2_, 21% O_2_). Cells were grown as described for panel (A) and then shifted to the indicated gas conditions. Growth was recorded after the shift to different gas conditions and back dilution to OD_730 nm_ ∼ 0.1. For panels (B) and (C), all cultivations were performed at a light intensity of 300 μE. The corresponding growth curves are shown in Supp. Figure S5.

### Growth Rate of *Synechocystis* with CbbM Is Dependent on the CO_2_/O_2_ Ratio in the Gas Feed

Contrasting to the *Synechocystis* Form I RbcLS, CbbM lacks the protective environment of carboxysomes. Carboxysomes are proteinaceous microcompartments which encapsulate Rubisco in close proximity to the enzyme carbonic anhydrase, and therefore help to elevate CO_2_ levels around Rubisco, as well as potentially lower the local O_2_ concentration.^38^ We confirmed the cytoplasmic localization of CbbM via fluorescence microscopy of a split-GFP fusion (Supp. Figure S2). Here, split-GFP was chosen to minimize potential disruption of CbbM dimerization. In addition, the lower abundance of several carboxysome constituents in RbcLS-depleted strains, as detected by untargeted proteomics (Supp. Note 1, Supp. Figure S3, and Supp. Table S5), indicates a general destabilization of the carboxysomes in these strains. Previous reports on the expression of the Type II Rubisco from also showed cytoplasmic localization.^33^

Since CbbM is localized in the *Synechocystis* cytoplasm, we assume that enzyme kinetic parameters will affect cellular growth rate depending on CO_2_ and O_2_ levels in the feed gas. We recorded batch cultivation growth curves of *Syn*-sgRNA­(−), *Syn*-sgRNA*_rbc_* and *Syn*-sgRNA_*rbc*_ *cbbM*(WT) in gas feeds with varying CO_2_/O_2_ ratios and determined specific growth rate (Figure 1C and Supp. Figure S5). Different gas feed compositions had only minor effects on the growth rates of *Syn*-sgRNA­(−) or *Syn*-sgRNA*_rbc_*. Carboxysome encapsulation of RbcLS in *Syn*-sgRNA­(−) likely protects the enzyme from changes in the exogenous gas feed. The slow growth of *Syn*-sgRNA*_rbc_* reflects the fact that RbcLS activity is essential for photoautotrophic growth of *Synechocystis*.^33,34^ Residual growth could result from a low remaining amount of RbcLS in the cell (Figure 1B).

At a high CO_2_/O_2_ ratio in the gas feed (5% CO_2_, 95% N_2_, 0% O_2_), expression of CbbM rescued the growth deficiency of *Syn*-sgRNA*_rbc_* and the strain *Syn*-sgRNA_*rbc*_ *cbbM*(WT) grew nearly as fast as *Syn*-sgRNA­(−) (Figure 1C and Supp. Figure S5). Because *Syn*-sgRNA_*rbc*_ *cbbM*(WT) harbors the pEEK plasmid and was grown with two antibiotics, while *Syn-sg*RNA­(−) does not harbor the plasmid and is grown with only spectinomycin, we tested whether the presence of chloramphenicol reduced the strain growth rate. We compared the growth rate of *Syn*-sgRNA_*rbc*_ *cbbM*(WT) with and without added antibiotics and found that the strain grew similarly in both cases (Supp. Figure S6). Therefore, the difference in growth rate is not due to the presence of additional antibiotics. Adding O_2_ (5% CO_2_, 75% N_2_, 20% O_2_) to the gas feed reduced the growth rate of *Syn*-sgRNA_*rbc*_ *cbbM*(WT), implying that the enzyme’s low specificity reduces the carboxylation rate at high O_2_ levels.

Proteomics analysis of *Syn*-sgRNA_*rbc*_ *cbbM*(WT) showed the upregulation of proteins related to CO_2_ uptake, such as SbtA, SbtB, and CupA compared to *Syn*-sgRNA­(−), even in a high-carbon feed gas 5% CO_2_ and 0% O_2_ (Supp Figure S3B). These genes are part of the NdhR regulon that mediates the response to low CO_2_, and indicates that the cell experiences carbon limitation in this condition (Supp. Table S6). The low CO_2_ response has previously been shown to be activated by Rubisco’s oxygenation product 2-PG.^38,39^ Therefore, it is possible that the O_2_ generated in situ by photosynthesis can lead to significant oxygenation activity by CbbM, even when the gas feed contains no O_2_.

When reducing the CO_2_ concentration in the gas feed to 1% CO_2_, 99% N_2_, 0% O_2_, the growth deficiency of *Syn*-sgRNA_*rbc*_ *cbbM*(WT) was slightly exacerbated, indicating that CbbM is operating below the limiting rate of Rubisco for CO_2_, *V*_C_, due to CO_2_ substrate limitation (Supp. Note 2). When adding oxygen to the 1% CO_2_ gas feed (1% CO_2_, 78% N_2_, 21% O_2_), the growth rate of *Syn*-sgRNA_*rbc*_ *cbbM*(WT) was reduced markedly. Thus, CbbM cannot support *Synechocystis* growth in a condition with 1% CO_2_, 78% N_2_, and 21% O_2_. The growth rates of *Syn*-sgRNA_*rbc*_ *cbbM*(WT) at different gas feed compositions are in line with those from a previous study where the *Synechocystis* RbcLS was exchanged for the Form II Rubisco.^33^

By changing the gas feed composition, our screening platform could be used to enrich CbbM variants that have improvements in different catalytic properties. A high CO_2_/O_2_ ratio in the gas feed (5% CO_2_, 95% N_2_, 0% O_2_), could be employed to enrich CbbM variants with a higher *V*_C_. At a lower CO_2_/O_2_ ratio, e.g. a gas feed of 5% CO_2_, 75% N_2_, and 20% O_2_, the specificity of a CbbM, should play a larger role in determining a strain’s growth rate, as the oxygenation reaction directs RuBP substrate away from the growth-benefiting carboxylation reaction. When lowering the substrate concentration to 1% CO_2_, the enzyme’s affinity for CO_2_, expressed by *K*_C_, should become more important for the growth rate.

Unlike carboxysome-located RbcLS, CbbM is dependent on cytosolic CO_2_ and might be substrate-limited even in a gas feed containing 5% CO_2_. We explored a strategy to increase the intracellular concentration of CO_2_ by deleting the NDH-1 subunits NdhD3 and NdhD4, encoded by genes *ndhD3* (*sll1733*) and *ndhD4* (*sll0027*), which abolishes the rapid transformation of cytosolic free CO_2_ into carbonate by the NDH-1 complex.^38,40^ The deletion did not increase the growth rate of *Syn*-sgRNA_*rbc*_ *cbbM*(WT) Δ*ndhD3* Δ*ndhD4* compared to *Syn*-sgRNA_*rbc*_ *cbbM*(WT) at gas feeds of 1 or 5% CO_2_ without added O_2_ (Supp. Figure S7). Increasing the CO_2_ concentration in the gas feed to 10% did not increase the growth rate of *Syn*-sgRNA_*rbc*_ *cbbM*(WT) to the level of wild-type *Synechocystis* (growth condition 10% CO_2_, 90% N_2_, 0% O_2_, Supp. Figure S8). It should be noted that a gas feed of 10% CO_2_ might be inhibitory to *Synechocystis* growth, as judged by the effect of high CO_2_ feeds on *Cyanothece* 51142 and PCC 11801.^41,42^ Alternative strategies to increase the strain’s growth rate are introducing the putative CbbM-specific Rubisco activase^43^ or by engineering a colocalization of CbbM and carbonic anhydrase, for instance in a carboxysome­(-like) structure.

### Phylogenetically Backed Strategy To Predict Beneficial Multisite Variants

In a next step, we wanted to test if the established screening platform could be used to find amino acid replacements that enhance CbbM activity. We worked from the assumption that multiple amino acid exchanges would be necessary to achieve an increase of activity. Furthermore, libraries of multisite variants could provide valuable information about epistasis between protein residues, making them better suited for exploring the protein fitness landscape of a protein than single-site variants.^44–46^ However, the chance of losing enzyme functionality increases with the extent of amino acid exchanges.^11–15^ To design a library of multisite mutants, we started with the zero-shot prediction method EVmutation. EVmutation uses multiple sequence alignments of homologues of the protein of interest to calculate the conservation of specific amino acids at each position, which results in an independent fitness prediction score.^17,18^ It then enriches this information by calculating the pairwise co-occurrence of amino acids at different positions, leading to an epistatic prediction score. The epistatic prediction score has shown to be more accurate than simple consensus prediction in predicting fitness of protein variants. Methods such as EVmutation capture evolutionary fitness as they are based on the idea that an amino acid that occurs in many homologues of a protein of interest likely conveys an evolutionary advantage, e.g., a higher stability, catalytic activity or an important protein interaction. This evolutionary fitness is distinct from the “organismal fitness,” of the individual *Synechocystis* mutant strains expressing a specific CbbM variant, which is dependent on CbbM kinetic parameters. However, previous work has shown that “evolutionary fitness,” is in many cases a good predictor of “organismal fitness” or fitness in some kind of assay to test for catalytic properties of an enzyme.^17,19^

To create the test library of CbbM, we aimed to test combinations of five amino acid exchanges predicted to impart higher fitness to CbbM, resulting in a library of in total 2^5^ = 32 different variants. To select five exchanges from the many possibilities, we used *in silico* evolution, which is a variant of the Metropolis-Hastings algorithm^20^ to heuristically explore the protein fitness landscape given by the EVmutation model (Methods, Supp. Table S8). Based on occurrence and co-occurrence frequencies during the *in silico* evolution, as well as predicted fitness values of the combinatorial variants, we decided on exchanges M140E, S162P, A230E, V323A and G422R (Figure 2B,C, Table 1, and Supp. Table S9). These were also among the ten replacements with the highest predicted fitness when introduced individually (Supp. Table S8) and, according to EVmutation, were not directly coupled among each other (Table 1, Supp. Figure S9). Indeed, the residues are located in different parts of the protein structure (Figure 2C). None of the amino acids was in close proximity to the enzyme’s catalytic center (roughly depicted by K200, a highly conserved lysine which is carbamylated during and essential for catalysis, shown in black in Figure 2C). Furthermore, all suggested residues faced away from the enzyme’s dimerization interface. Exchanges were therefore assumed to not affect dimerization or RuBP binding directly. All suggested exchanges except V323A were surface-exposed.

**Figure 2:**
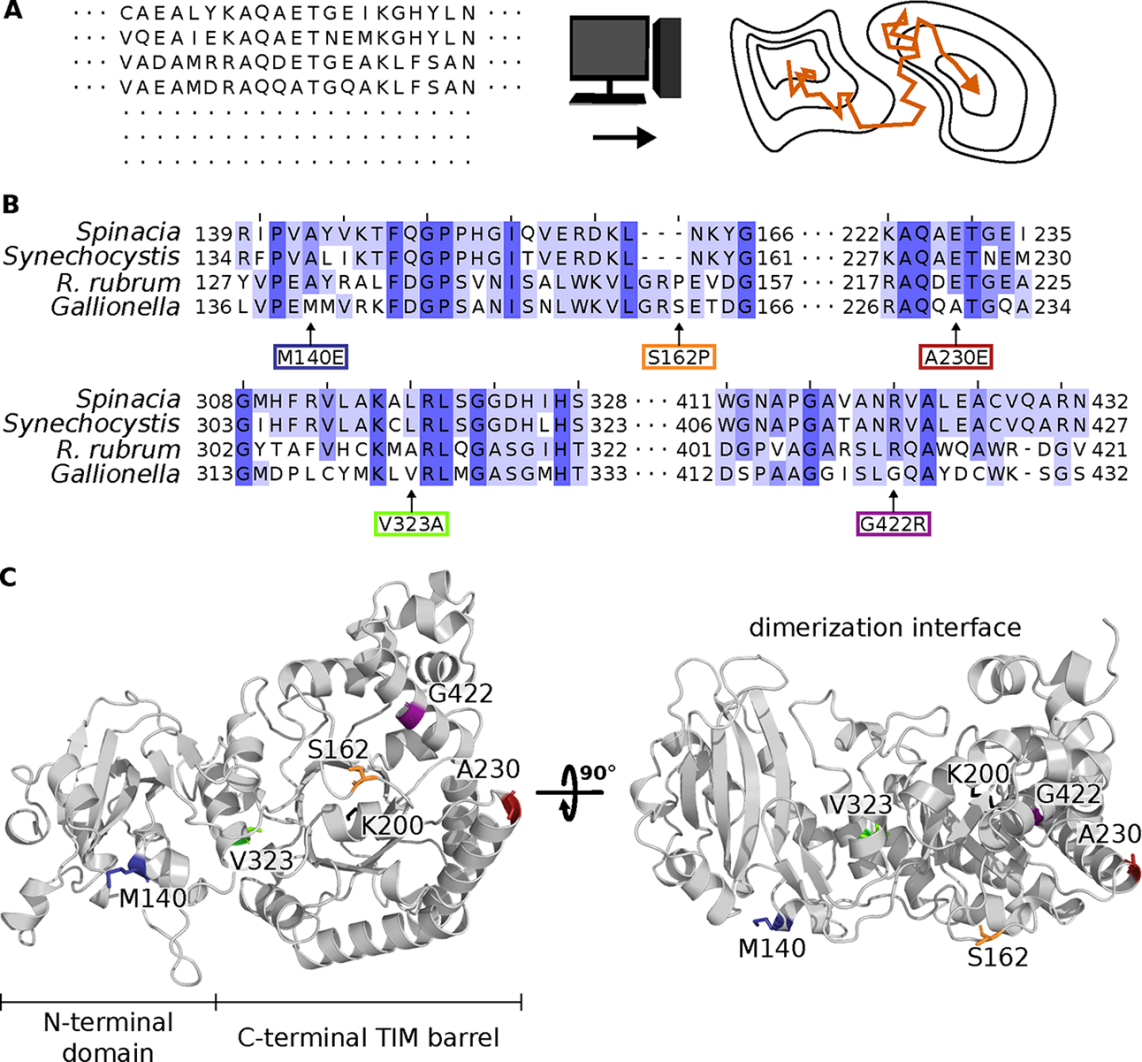
Phylogenetically backed strategy to predict beneficial amino acid exchanges in *Gallionella* CbbM. (A) Graphical depiction of the EV couplings and *in silico* evolution used to design CbbM mutants. The sequence of *Gallionella* sp. CbbM/Rubisco (GenBank identifier OGS68397.1) was used as the starting point to create a multiple sequence alignment using the EVcouplings web server. The resulting MSA with 1905 sequences and the EVcouplings model was used to perform *in silico* evolution and identify new local fitness maxima in order to suggest beneficial *cbbM* variants. *In silico* evolution describes a heuristic algorithm to identify local fitness maxima by traversing “fitness valleys” between different “fitness maximum peaks”. (B) Alignment of fragments of the amino acid sequences of well investigated Rubisco homologues of (*Spinacia*, P00875) and (, P04718), as well as *Synechocystis* sp. PCC 6803 (*Synechocystis*, P54205) and *Gallionella* sp. (*Gallionella*, A0A1G0AW29). Arrows point at the amino acid exchanges highlighted in (C). Shades of blue indicate different degrees of identity between the compared protein sequences. Numbers give the amino acid position of the first and last amino acid shown in the alignment. (C) Structure of *Gallionella* Rubisco as cartoon model, on the left shown from the side which is facing away from the dimerization interface. On the right, the structure is shown turned by 90°. Amino acid positions suggested to be exchanged and carbamylated lysine K200 are highlighted in color and shown as stick models.

**Table 1:** Amino Acid Exchanges Suggested by EVmutation Coupled to Metropolis-Hastings Algorithm^a^ sifile2

amino acid exchange	EVmutation (independent)	EVmutation (epistatic)	coupled to
M140E	4.2	2.6	K22, V96
S162P	1.2	3.1	W156
A230E	2.5	2.9	Y190, L194, E258
V323A	1.1	2.8	G275, P280, F297
G422R	6	4.7	Q233

a EVmutation independent and epistatic fitness predictions are given. Epistatic fitness predictions take evolutionary couplings into account, whereas independent scores are only based on occurrence frequencies. Column “coupled to” gives residues that are predicted to be evolutionary coupled to the respective position. For fitness predictions of all possible amino acid exchanges covered by the used MSA compare Supp. Table S8.

It is noteworthy that all suggested exchanges except M140E changed the CbbM residue to that present in the Rubisco at the corresponding position (Figure 2B, amino acid/*Gallionella* amino acid: P153/S162, E221/A230, A312/V323, R411/G422). Nevertheless, we checked the approximate effects of exchanges at the suggested positions according to a recently published DMS of Rubisco.^10^ Most exchanges at these homologous sites of the suggested positions did not show a strong effect on catalytic parameters in Rubisco. However, amino acid exchanges may show sign epistasis when incorporated into a different sequence background.^12,44,45^

We designed double, triple, and quadruple-site CbbM variants containing combinations of each of these exchanges. Each variant was tagged with a nontranslated 20 nt barcode, which enabled tracking of variant abundance by Illumina sequencing. We were not able to obtain any transformants when creating the M140E variant. Hence, we continued with a 16-member library based on the remaining four amino acid exchanges. The EVmutation fitness values for these 16 variants are given in Supp. Table S9.

### In Vivo Pooled Screening Identified Epistasis among Amino Acid Exchanges

We performed pooled growth competition assays at different gas feeds and light conditions (Figure 3, Supp. Figures S10–S12, and Supp. Table S10). Based on our pre-experiments (Figure 1C), we included growth at continuous light of 300 μE and a gas feed of 5% CO_2_ with or without added oxygen (5% CO_2_, 95% N_2_, 0% O_2_ and 5% CO_2_, 75% N_2_, 20% O_2_). For both conditions, we ran four technical replicate cultivations, which were inoculated from the same mutant pool. The different CbbM variants were detected in similar amounts prior to the start of the growth competition experiments (gini index for all samples of the original pool <0.15). The replicates of both conditions showed a high correlation per time point, as measured by Pearson’s *r* (*r* > 0.8 among replicates for 80% of samples).

**Figure 3:**
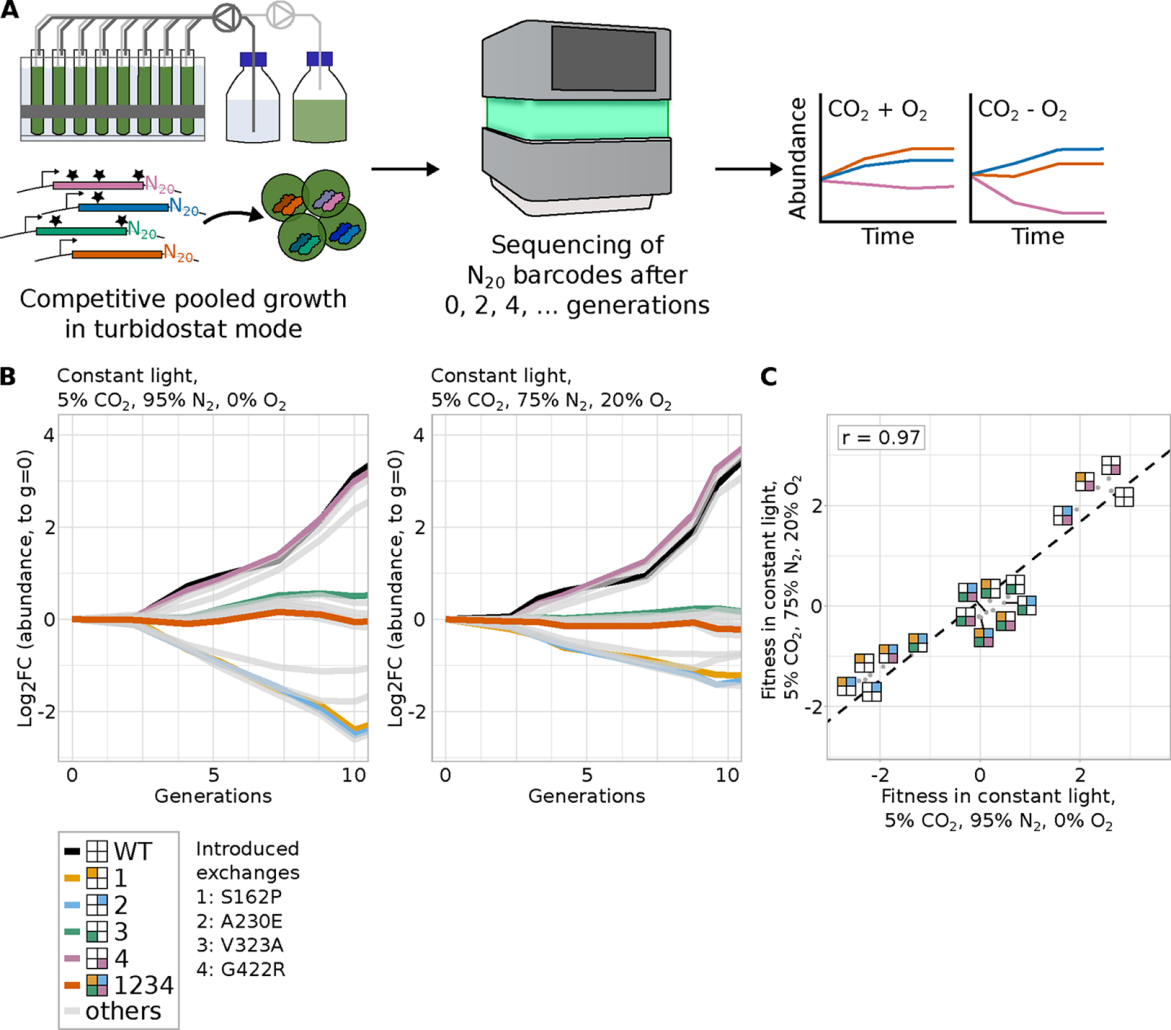
In vivo screening of library of Rubisco variants. (A) Scheme illustrating high-throughput screening by competitive growth of the pooled variant library (left) followed by next generation sequencing (middle) and fitness calculations (right). (B) Changes of abundance of different strains during competitive growth of the 16-member library at different growth conditions. For reasons of clarity, data of only the wild-type variant, variants with a single amino acid exchange and of the quadruple mutant variant are depicted in color. Fitness values of other variants are plotted in light gray. For a plot including 95% confidence intervals of the highlighted variants, see Supp. Figure S11A. Fitness data of all variants is shown in Supp. Figure S11B. Light intensity for all cultivations was 300 μE. (C) Scatter plots comparing the fitness data from different growth conditions. The Pearson correlation coefficient r is given for the comparison of fitness values at the two different continuous light conditions (*r* = 0.97, *p* = 1.833 × 10^–10^). Panels (B) and (C): For reasons of clarity, we depict every variant as consisting of four boxes which represent the four different introduced amino acid exchanges. If a box is colored, the exchange was introduced and if left white, the exchange is absent.

Fitness values of different mutant variants were determined based on the increase or decrease in relative abundance over time. Fitness values for each mutant were highly correlated between both growth conditions (Figure 3C), implying that none of the variants changed CbbM specificity significantly. No mutant variant gave a significantly higher fitness value than the wild-type CbbM, with three variants being on par with it. Fitness values separated into three groups (Figure 3B,C). The fitness value distribution revealed a small epistatic network among the tested amino acid exchanges (Figures 3C and 4A). In the following, to make naming of multisite variants easier, we will refer to the different replacements by numbers based on their relative position in the protein’s coding sequence: 1: S162P, 2: A230E, 3: V323A, 4: G422R. For instance, we will refer to the variant with S162P, V323A and G422R as CbbM(134). Note that amino acid numbering refers to the wild-type CbbM sequence excluding the 14 amino acid Strep tag. Amino acid exchanges S162P and A230E were detrimental to growth (Figure 3C). Single-site variants CbbM(1) and CbbM(2) and the combined variant CbbM­(1,2) were the slowest growing mutants. The slow growth of variant *Syn*-sgRNA_*rbc*_ *cbbM*(12) was confirmed by cultivation of the axenic strain in batch; the strain grew nearly as slow as *Syn*-sgRNA*_rbc_* which lacked *cbbM* complementation (Figure 1C and Supp. Figures S5, S13). Based on Western blot analysis of these variants (Figure 4B), the growth deficiency was based on a strongly reduced CbbM expression of variant CbbM(12) compared to the wild-type variant CbbM­(WT) or variant CbbM(14). Hence, the exchange A230E had a negative effect on CbbM expressibility, solubility or stability.

**Figure 4:**
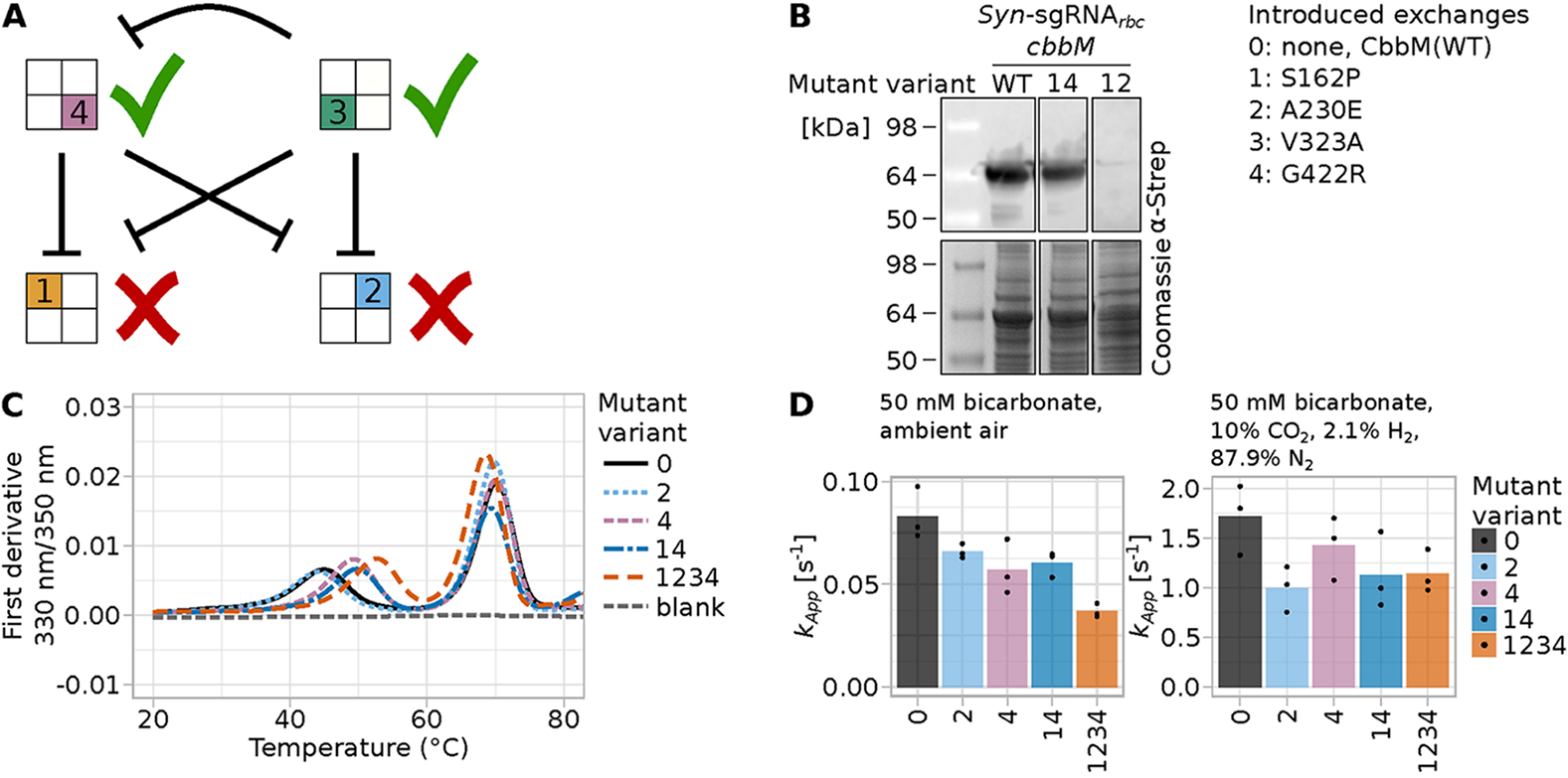
Epistasis among introduced amino acid exchanges. In all panels, introduced exchanges are coded as 1: S162P, 2: A230E, 3: S162P, 4: G422R. (A) Scheme illustrating epistasis of different mutations. For reasons of clarity, we depict every variant as consisting of four boxes which represent the four different introduced amino acid exchanges. If a box is colored, the amino acid exchange was introduced. If left white, the amino acid exchange is absent. (B) Western blot analysis (upper panels) and Coomassie stain (lower panels) of the soluble fraction of *Synechocystis* cell lysates of strains expressing selected variants to determine the mutant protein’s abundance. For detection, horseradish peroxidase (HRP)-conjugated antibody directed against the encoded N-terminal Strep tag (αStrep) was used. HRP chemiluminescence signal is overlaid with an image of the membrane. See Supp. Figure S14 for the complete blot and Coomassie-stained SDS-PAGE gel. The protein of interest, N-Strep-tagged CbbM, has a size of 53 kDa. (C) Thermal melting curves of several CbbM variants expressed in and purified from as determined by nanoDSF (compare Supp. Table S11). (D) Apparent rate constants as measured by spectrophotometric in vitro assays. The assays were conducted either in ambient air (approximately 0.04% CO_2_, 78% N_2_, 21% O_2_) or in a gas atmosphere without oxygen (10% CO_2_, 2.1% H_2_, 87.9% N_2_, 0% O_2_). In both cases, 50 mM bicarbonate was added to the reaction mixture.

All variants which included the exchange V323A, and which were not part of the first described group, formed a group which grew approximately at the pool’s average growth rate (Figure 3B,C). The last group contained wild-type CbbM (strain *Syn*-sgRNA_*rbc*_ *cbbM*(WT)) as well as strains containing the exchange G422R and either S162P or A230E, but not both: CbbM(4), CbbM­(1,4), CbbM­(2,4). These four strains outperformed all other strains. We confirmed the comparable growth rates of *Syn*-sgRNA_*rbc*_ *cbbM*(14) to *Syn*-sgRNA_*rbc*_ *cbbM*(WT) in axenic cultivations (Supp. Figure S13). According to Western blot analysis, soluble CbbM levels in both strains were very similar (Figure 4B and Supp. Figure S14). Hence, the exchange G422R rescued the detrimental effects of S162P and A230E, but had no positive effect when introduced on its own. Interestingly, introducing the exchange V323A in these variants reduced fitness, showing that V323A was epistatic to G422R (Figure 4A).

### Long-Distance Interactions between Tested Amino Acid Positions

Rubisco fitness as measured in our screening platform did not correlate with the predicted values given by EVmutation (Supp. Figure S15A) nor with the total number of exchanges introduced compared to the wild-type CbbM (Supp. Figure S15B). In contrast to EVmutation predictions (Table 1), and despite their spatial separation in the enzyme, the investigated amino acid exchanges showed epistasis among each other. Similar long-range amino acid interactions were also found for other proteins^44,46,47^ and also specifically for Rubisco homologues.^6,48–50^ EVmutation captures evolutionary pressures on the enzyme in nature, which does not necessarily correlate with the pressures exerted in the concrete experimental system under investigation. Alternatively, correlation between EVmutation predictions and fitness values might only be observable when investigating a larger set of variants. All suggested exchanges except for M140E, which could not be expressed in , corresponded to the Rubisco sequence (Figure 2B). A possible explanation might be biases present in the sequence database used for the construction of the multiple sequence alignment,^51^ which might reduce the predictive power of EVmutation and similar algorithms. This highlights the need for further metagenomic screenings and the characterization of phylogenetically more diverse variants to capture the full diversity and potential of Rubisco (compare^8^).

### In Vitro Characterization Shows CbbM Expression, Stability, and Activity Changes Underlie In Vivo Growth Differences

We characterized a small subset of the CbbM variants in vitro after purification from (Figure S16). Protein melting temperature was measured using nanoDFS and showed two distinct melting points for all investigated variants (Figure 4C, Supp. Table S11). The two melting points of CbbM­(WT) were approximately 45 and 70 °C. These were only minimally affected by the introduction of A230E in variant CbbM(2). The lower melting point increased to approximately 49 °C for variants CbbM(4) and CbbM(14), hinting toward a stabilizing effect of exchange G422R. This was raised even further, to approximately 52.6 °C, by the introduction of either S162P and/or V323A in variant CbbM(1234). However, the second, higher melting point was slightly lowered in CbbM(1234) compared to all other variants. Hence, it is not clear if S162P and/or V323A might stabilize parts of the protein while destabilizing others.

We used a spectrophotometric in vitro assay to test for differences in kinetic enzyme parameters among selected CbbM mutants (Figure 4D, Supp. Figures S16, S17). We performed the assay in a high CO_2_, anaerobic condition intended to mimic the in vivo fitness screen condition, as well as an ambient air condition. In the high CO_2_ condition, the calculated apparent rate constant of the CbbM variants reflected to some degree the relative fitness scores from the in vivo competition experiments, with CbbM(2) having the lowest apparent rate constant (Figure 4D). Hence, changes in enzyme parameters *k*_cat,C_ or *K*_C_ may also contribute to the observed growth differences, in addition to changes in protein stability and solubility. In ambient air condition, which was not used for fitness screening, the relative ranking of enzyme rate constants was different.

### Establishment of a Photoautotrophic High-Throughput Rubisco Screening Platform

In conclusion, our established screening system enabled us to test a pool of multisite CbbM variants in parallel in vivo in a photoautotrophic host. The CbbM from *Gallionella* has the highest *k*_cat,C_ reported among all Rubiscos. Replacement of the native Form I Rubisco with CbbM results in a Rubisco outside the carboxysome and thus the resultant strain becomes high CO_2_ requiring. Furthermore, the proteomic response suggests that in situ O_2_ produced from photosynthesis can be a significant source of oxygenation of RuBP by the enzyme, even in an anoxic gas feed. Future work could quantify the photorespiratory metabolites. Nonetheless, the CbbM strain grew rapidly at high CO_2_.

The coupling of EVmutation with *in silico* evolution is intended to reduce the vast number of possibilities in a combinatorial protein library to a smaller set that is more likely to improve fitness. The experimental screening system is analogous to CRISPRi or BarSeq screens previously established in cyanobacteria, where the composition of a mutant population of thousands of members are tracked during growth competition in different conditions.^34,52^ Even the small pool of variants designed and tested here show the utility of applying barcoded genetic screens for exploring a protein fitness landscape. Two of the amino acid exchanges suggested by EVmutation, G422R and V323A, helped to rescue the detrimental effect of the other two exchanges and may also contribute more generally to an increased robustness against detrimental replacements in the protein. Furthermore, G422R and all variants containing it had increased thermal stability. Without a controlled comparison to random combinatorial mutagenesis, it is difficult to know if the mutations suggested by EV mutation and *in silico* evolution are enriched for exchanges that are involved in epistasis.

The scalability of a barcode in vivo screening system also allows testing of Rubisco fitness in other cultivation conditions relevant for photosynthetic metabolism, such as lower CO2 feeds, mixotrophic growth with added sugar, or growth in diurnal light cycles. Such conditions could be expected to impart different selection pressures on Rubisco function and sequences, due to their known impact on metabolite levels.^27–30^

## Methods

### Prediction of Beneficial Multisite Mutant Variants Using EVmutation and In Silico Evolution

The amino acid sequence of *Gallionella* Rubisco, CbbM, (GenBank identifier OGS68397.1)^53^ was used as input for the EVmutation^17,18^ web server (https://v2.evcouplings.org/) with default settings (bitscores 0.1, 0.3, 0.5, 0.7; 5 search iterations; sequence database UniRef90;^54^ position filter 70%; sequence fragment filter 50%; removing similar sequences 90%; downweighting similar sequences 80%; statistical inference model: pseudolikelihood maximization). The web server identified a bitscore of 0.5 as optimal for alignments, which led to a multiple sequence alignment of 1905 sequences (*N*_eff_/*L* = 4.9). The created model and multiple sequence alignment were downloaded and the inferred fitness landscape was navigated using *in silico* evolution, i.e., a Metropolis-Hastings Monte Carlo Markov chain,^20^ to identify multisite mutant variants with predicted fitness values higher than the wild-type sequence. We decided to use this algorithm to explore the fitness landscape given by the Potts model created by EVmutation, but it can also be combined with other protein fitness prediction models, e.g., DeepSequence. The used code is available on https://github.com/ute-hoffmann/EVmut_inSilico and Zenodo (doi: 10.5281/zenodo.13971225). Briefly, an *in silico* evolution trajectory consists of multiple mutation steps. The algorithm started with the wild-type protein sequence of CbbM as the initial state. In each following step, a random amino acid exchange was proposed and the fitness value of the resulting sequence was predicted by EVmutation. Otherwise, with a certain acceptance probability given by the temperature value, the step was accepted nonetheless. Hence, the algorithm can help to traverse valleys of protein sequences with low predicted fitness values and escape local fitness maxima. This would not be possible in the case of a greedy algorithm that only accepts proposed sequences with increased fitness values. A trust radius determined how many amino acids could be exchanged in total. To determine amino acid positions of interest, 2000 trajectories were run with 500 steps each and a trust radius of two for three different temperatures (0.3, 0.03, 0.003). Based on these results, a temperature of 0.03 was used for further analyses. The sequences with the highest predicted fitness values were extracted from the trajectories and the introduced amino acid exchanges were identified and counted. The 30 most frequently occurring replacements were used for another run of *in silico* evolutions, in which possible amino acid exchanges were limited to these respective positions. In this second run, 750 trajectories were run with 500 steps and a trust radius of three. The amino acid exchanges which were co-occurring most frequently among the highest scoring sequences were picked for library construction.

For visualization of the amino acid exchanges of interest, we used the AlphaFold 2.0 structure prediction^55,56^ of CbbM (https://alphafold.ebi.ac.uk/entry/A0A1G0AW29) and the PyMOL Molecular Graphics System, Version 2.5.0 (Schrödinger, LLC.). Multiple sequence alignments were created using Clustal Omega provided by EMBL-EBI^57^ and analyzed using Jalview (v2.11.0).^58^ Figures were created using Inkscape (v1.1).

### Bacterial Strains and General Culture Conditions

We used a nonmotile glucose-tolerant wild type of *Synechocystis*, which was kindly provided by Pauli Kallio, University of Turku. For culturing, BG-11 medium^59^ supplemented with HEPES buffer (20 mM, pH 7.8) was used. If not cultivated in a photobioreactor or indicated otherwise, cultures were grown at 30 °C under continuous white-light (45 μmol photons m^–2^ s^–1^) in ambient air enriched with 1% (v/v) CO_2_. Liquid cultures were grown in flat base uncoated 6-well plates (Sarstedt) or Erlenmeyer flasks under constant shaking (160 rpm). Plate cultures were grown on 1% (w/v) bacto-agar BG-11 plates containing 0.3% (w/v) sodium thiosulfate. Kanamycin (50 μg mL^–1^), spectinomycin (30 μg mL^–1^), gentamicin (2 μg mL^–1^), erythromycin (20 μg mL^–1^), and anhydrotetracycline (aTc, concentrations as indicated) were added to plate and liquid cultures when needed. For construction of plasmids carrying the *SPdcas9* gene, the CopyCutter EPI400 (Biosearch Technologies (Lucigen)) cell line was used. For all other cloning, the XL1-Blue cell line was used. For protein expression, BL21­(DE3) were used. When appropriate, kanamycin (50 μg mL^–1^) was added to cultivation media.

### Construction of Mutant Strains

All *Synechocystis* strains and relevant oligonucleotides used in this study are listed in supplementary Tables S12 and S13. Annotated sequences of plasmids used for strain engineering are provided in .gb file format as Supporting Information. All RSF1010-derived pEEK2-based plasmids^60^ were transferred into receiver *Synechocystis* strains by triparental mating with XL1-Blue harboring the constructed plasmids and HB101 helper cells with the plasmid pRL443-Amp^R^ as described in.^60^ All other plasmids were introduced into the respective receiver strains by natural transformation.

All cloning was done by a combination of PCR, restriction enzyme digestions, ligations and AQUA cloning.^61^ Plasmids used for the insertion of a partial or complete CRISPRi system were derived from the plasmid pMD19T_psbA1_PL22_dCas9_B0015_SpR,^35^ which introduces the gene encoding catalytically dead Cas9 (dCas9, mutations D10A and H840A) from , *SPdcas9*, as part of the tetR_PL22_dCas9_SpR expression cassette into the *slr1181* locus (*psbA1*). The annotated DNA sequence of the resequenced plasmid is given in file dcas-empty.gb. For targeting *rbcL* using CRISPR inhibition, the two protospacers with the lowest fitness values under highlight, high-CO_2_ (HL HC) and lowlight, low-CO_2_ (LL LC) conditions in a recent CRISPRi screen^34^ were chosen: rbcL|10 (5′-ACGCCCGCCTTAAACCCTGCTT-3′) and rbcL|47 (5′-TCGGGGGTATAGTAGGTC-3′). To create the full CRISPRi system, both were placed under the control of an anhydrotetracycline (aTc)-inducible L22 promoter.^62^ The DNA sequence of the plasmid introducing dCas9 and these sgRNAs is given in file dcas9_2xsgrna.gb. Before using the strain carrying the complete CRISPRi construct for downstream strain construction, a homozygous insertion of the construct into the genome was checked by cPCR using a small amount of cell material as template, DreamTaq 2X Green PCR Master Mix (Thermo Scientific) and primer pairs P01/P02 as well as P03/P04 (Supp. Figure S18).

*Gallionella* Rubisco (GenBank identifier OGS68397.1)^53^ was codon-optimized for expression in *Synechocystis* and synthesized as gBlocks gene fragment (idt). All *Gallionella* Rubisco variants were expressed from RSF1010-derived pEEK2-based plasmids under the control of the constitutive trc promoter. Point mutations were introduced into the *Gallionella* Rubisco coding sequence by performing PCRs using Phusion DNA polymerase (Thermo Scientific) and primer pairs M01/M02, M03/M04, M05/M06, M07/M08 and M09/M10, which were designed using NEBaseExchanger (NEB). Subsequently, 1 μL PCR product was mixed with 1 μL T4 DNA ligase buffer (10×), 1 μL T4 PNK (10 U), 1 μL T4 DNA ligase (5 U), 1 μL DpnI (10 U) (all Thermo Scientific) and 5 μL H2O, incubated for 1 h at room temperature and transferred to XL1-Blue. Higher order mutant variants were obtained by running several rounds of site-directed mutagenesis on the isolated plasmids of respective lower order mutant variants. Mutant variants were tagged with individual 20 nucleotide long barcodes which had been ordered as DNA fragments from idt.

The split GFP system was developed to minimize the potentially negative impact of GFP-tagging on a protein of interest.^63^ To this end, sfGFP was split into two parts and optimized for optimal assembly. DNA fragments with matching overhangs and encoding either of the two parts, i.e. the 11th α helix or alpha helices 1 to 10 of GFP, were ordered from idt and used to tag N-Strep-tagged CbbM C-terminally with GFP11 and create a plasmid integrating the gene encoding spGFP1–10, *spGFP1–10*, at the locus of *slr0168* under the control of a rhamnose-inducible promoter. The DNA sequence of the plasmid introducing the gene encoding Strep- and GFP11-tagged CbbM can be found in the file peek2-cbbm-gfp11.gb. For introducing either the rhamnose-inducible spGFP1–10 construct or, as a control, only a chloramphenicol resistance cassette, plasmids NS-GFP10 and sp3-CmR were created. Annotated DNA sequences of both plasmids can be found in files ns-gfp10.gb and sp3 cmr.gb.

Genes *ndhD3* and *ndhD4* were deleted using natural transformation (ndhd3_deletion_plasmid.gb and ndhd4_deletion_plasmid.gb). Full segregation of the resulting strains was tested by PCR using primer pairs P05/P06 for *ndhD3* and P07/P08 for *ndhD4* (Supp. Figure S18) on DNA extracted using the GeneJET Genomic DNA Purification Kit (Thermo Scientific) according to the manufacturer’s Gram-positive extraction protocol.

### Microscopy

Liquid cultures of *cbbM*-GFP_11_|GFP_1–10_ and the control strain *cbbM*-GFP_11_|noGFP were diluted to an OD_750 nm_ of 0.4 and expression of GFP_1–10_ was induced by the addition of rhamnose (4 mg mL^–1^) in one replicate of each culture. When cultures reached an OD_750 nm_ of approximately 1.7, 2 mL of each culture and condition were harvested by centrifugation (6000*g*, 5 min, 20 °C). Supernatant was carefully decanted and pellets were resuspended in approximately 50 μL BG-11. Of these, 5 μL were spotted on a 1.8% (w/v) agarose (in phosphate-buffered saline) plate. These spots were dried for 10 to 30 min in a laminar flow cabinet. Spots were excised using a scalpel and inverted onto an uncoated 35 mm diameter Petri dish with 20 mm diameter No. 1.5 glass bottom (MatTek). Confocal imaging was performed using a Zeiss LSM780 confocal microscope equipped with a 40×/1.2 NA water immersion objective and a spectral detector. GFP fluorescence was excited using a 488 nm laser, with emission captured between 493 and 630 nm. Autofluorescence was excited with a 633 nm laser, and emission was detected from 638 to 755 nm. The pinhole size was set to 1 Airy unit for both channels to ensure optimal optical sectioning and to maximize the signal-to-noise ratio. Images were analyzed using (Fiji Is Just) ImageJ 1.54f.^64^

### Cultivation in Photobioreactors

If indicated, cultivations in 8-tube Multi-Cultivator MC-1000-OD bioreactors (Photon System Instruments, Drasov, CZ) were performed using the pycultivator-legacy package (https://gitlab.com/mmp-uva/pycultivator-legacy)^65^ and as described^34^ with the following modifications: Light intensity was kept at 300 μmol photons m^–2^ s^–1^. If run in turbidostat mode, cultures were diluted if the turbidity threshold of OD_720 nm_ = 0.2 was exceeded for two measurements in a row. Cultures were grown from the beginning of the cultivation at the indicated gas mixture. To create the pooled CbbM library, 16 different *Synechocystis* transformations were performed (one for each CbbM variant). Then, from each transformation plate, three colonies were picked and grown up separately, except for strains with variant CbbM­(S162P, V323A, G422R), for which only two transformants were obtained and strains with variants CbbM­(S162P), CbbM­(V323A, G422R), and CbbM­(S162P, G422R) where only a single transformant colony was obtained. Liquid from each of the cultures was then combined to create the pooled library. This pooled library was then split into eight separate culture tubes (4 for 5% CO_2_ anoxic and 4 for 5% CO_2_ air condition) in preparation for growth competition experiments. For growth competition assays of this pool, CRISPRi repression was induced by adding aTc (0.2 μg mL^–1^) to cultivation tubes and feeding bottles after pools had acclimated and stable cultivation with regular back-dilutions was reached. Addition of aTc marked generation 0. After 24 h, the aTc concentration was raised to 1 μg mL^–1^. Growth data was analyzed using the ShinyMC web application (https://github.com/m-jahn/ShinyMC, v0.1.1). Growth rates were calculated with “OD correction” set to true. The average growth rate of replicates was used for calculation of doubling times, i.e., the duration of one generation *t*, using the formula $t=\frac{\ln(2)}{\mathrm{growth}\;\mathrm{rate}}$ . Cells were harvested after approximately the second, fourth, sixth, eighth, and 10th generation by centrifuging 12 mL culture (4500*g*, 4 °C, 10 min). Supernatant was removed completely and cell pellets were frozen at −20 °C until further usage for NGS preparation.

For batch cultivations of strains *Syn*-sgRNA­(−), *Syn*-sgRNA*_rbc_* and *Syn*-sgRNA_*rbc*_ *cbbM*(WT) at different gas conditions, precultures were grown in ambient air enriched with 1% (v/v) CO_2_. For strains *Syn*-sgRNA­(−) and *Syn*-sgRNA*_rbc_*, plate cultures of a single transformed *Synechocystis* colony were prepared in advance. For strain *Syn*-sgRNA_*rbc*_ *cbbM*(WT), three independent clones from a single transformation were used for experiments. After inoculation from plate cultures, liquid cultures were grown to an OD_730 nm_ of approximately 0.8, diluted to an optical density OD_730 nm_ = 0.2 and grown to OD_730 nm_ = 0.8 to ensure a similar growth state of all used cultures. Subsequently, cultures were diluted to 0.2 and aTc (0.5 μg mL^–1^) was added. When cultures reached OD_730 nm_ = 0.8, they were diluted to 0.1, aTc (0.5 μg mL^–1^) was added, and transferred into the bioreactors at the indicated gas feed conditions. Additional aTc (0.25 μg mL^–1^) was added every second day. For mass spectrometric analyses, 12 mL of culture was collected by centrifugation (4500*g*, 4 °C, 10 min) when an optical density of OD_720 nm_ = 0.8 was reached.

For batch cultivations of strains *Syn*-sgRNA_*rbc*_ *cbbM*(WT), *Syn*-sgRNA_*rbc*_ *cbbM*,^12^ *Syn*-sgRNA_*rbc*_ *cbbM*,^14^ wild-type *Synechocystis* and Δ*ndhD3* Δ*ndhD4* strains, precultures were grown at a gas feed of 5% (v/v) CO_2_, 75% (v/v) N_2_, 20% (v/v) O_2_ and a light intensity of 50 μE. Liquid cultures were diluted to OD_730 nm_ = 0.4 and aTc added (1 μg mL^–1^). After 48 h, this was repeated. After another 48 h, cultures were diluted to OD_730 nm_ = 0.05 and transferred to photobioreactors at the indicated gas feeds.

Growth rates of batch cultures in photobioreactors was determined by performing a linear regression between 12.5 and 37.5 h after transferral of cultures to the bioreactors using raw OD values and batch time as *y* and *x* values, respectively. One replicate of *Syn*-sgRNA_*rbc*_ *cbbM*(WT) was excluded from analyses at 1% (v/v) CO_2_, 78% (v/v) N_2_, 21% (v/v) O_2_, since its gas feed had stopped. Same holds for all replicates of wild-type Synechocystis at a gas feed of 1% (v/v) CO_2_, 99% (v/v) N_2_ and 0% (v/v) O_2_.

### Library Preparation and Next-Generation Sequencing

The GeneJET Plasmid-Miniprep-Kit (Thermo Scientific) was used to extract plasmids from cell pellets collected from turbidostat cultivations. Cell pellets were resuspended in 250 μL resuspension buffer and mixed with 50 μL of glass beads (diameter 425–600 μm, Sigma-Aldrich) in 1.5 mL protein LB SC Microtubes (Sarstedt). Samples were homogenized using a FastPrep-24 5G bead beating grinder and lysis system (MP Biomedicals) in three cycles of 45 s at 6.5 m s^–1^ with 30 s on ice between cycles. The plasmid extraction was continued according to the manufacturer’s protocol. DNA was eluted with 20 μL deionized 55 °C-warm water. To amplify the barcode region of the plasmids by PCR, 9 μL of DNA eluate were used as template in a reaction volume of 30 μL using NEBNext Ultra II Q5Master Mix (NEB) and PAGE-purified oligonucleotides S1 and an equimolar mixture of S2, S3, and S4 as forward and reverse primer, respectively. Oligonucleotides S2, S3, and S4 only differed regarding the length of a short stretch of random nucleotides (N, NN, NNN). Mixing the three oligonucleotides enabled phasing and to increase diversity in the resulting sequencing library. The manufacturer’s protocol for NGS PCRs and six elongation cycles were used. The PCR products were purified using Agencourt AMPure XP (Beckman Coulter, Inc.) according to manufacturer’s protocols and eluting DNA using 20 μL deionized water. In a second PCR, indices for multiplexing were added with the same PCR conditions as described above and using different combinations of oligonucleotides from the single and dual index sets of NEBNext Multiplex Oligos for Illumina (NEB) as primers. The amount of PCR product was quantified using a Qubit dsDNA HS Assay Kit and a Qubit 4 Fluorometer (both Thermo Scientific). Equal amounts of each sample were pooled and PCR product of the expected size was purified by performing a gel extraction from a 1.8% (w/v) agarose gel (in Tris acetate EDTA buffer, stained with Serva Electrophoresis DNA Stain G (Serva Electrophoresis)) using the GeneJET Gel extraction kit (Thermo Scientific) according to manufacturer’s instructions, except that elution was performed using 12 μL deionized 55 °C-warm water. The concentration of PCR product was quantified using a Qubit TM 4 Fluorometer (Thermo Scientific) as described above and used for sequencing on a NextSeq 2000 system (Illumina) using NextSeq 1000/2000 P2 reagents v3 (Illumina) according to the manufacturer’s instructions.

### Analysis of Sequencing Data

Sequencing data was analyzed as described^34^ by using the Nextflow pipeline (https://github.com/MPUSP/nf-core-crispriscreencommite4aad5be10264d99e632761fc8fc56e68d6357c4 from 11th April 2024). Sequencing data was mapped to a library of the used barcodes. Compared to default settings, the error rate (parameter error_rate) was set to 0.2, the mapping quality cutoff (parameter filter_mapq) set to 1 and the sequence GTCTAGAatcgccgaaagtaattcaactccattaa···TCTAGATGCTTACTAGTTACCGCGGCCA was used for adapter trimming (parameter five_primer_adapter). Parameters run_mageck and gene_fitness were set to false. Further analyses were performed in the R programming language and are documented in R Markdown notebooks available at https://github.com/ute-hoffmann/CbbM_16variants (Zenodo doi: 10.5281/zenodo.14699592).

### Proteomics Sample Preparation

Cell pellets were collected as described for photobioreactor batch cultivations. Pellets were resuspended in 200 μL lysis buffer (100 mM HEPES pH 8, 1.5 M KCl, 3 mM MgCl) containing protease inhibitors (cOmplete, EDTA-free Protease Inhibitor Cocktail, Roche) and mixed with 100 μL glass beads (diameter 425–600 μm, Sigma-Aldrich). They were lysed by bead beating (FastPrep-24 5G lysis machine, MP Biomedicals) over six cycles of 45 s at 6.5 m s^–1^ and 4 °C, with 30 s breaks on ice between cycles. The samples were then pelleted by centrifugation (21,000*g*, 5 min, 4 °C) and supernatants were transferred to new tubes. Protein concentrations in the lysates were measured using a bradford assay (Bio-Rad protein assay dye reagent) using BSA as a standard at concentrations of 0, 0.05, 0.1, 0.2, and 0.4 mg mL^–1^. Lysates were diluted to a protein concentration of 2 mg mL^–1^ before being reduced with 18 mM DTT for 10 min at 96 °C. Samples were then alkylated with 10 mM IAA for 30 min at 25 °C before being diluted 4× in 100 mM ammonium bicarbonate. Subsequently, samples were digested by a trypsin/LysC protease mix (Pierce Trypsin/LysC protease mix, MS grade, Thermo Scientific) at a protein to protease mass ratio of 50:1 for 16 h at 37 °C with 600 rpm of shaking, after which they were quenched with formic acid to pH < 2 and centrifuged (21,000*g*, 10 min, 25 °C). Supernatants were then desalted through stage tips packed with six layers of C18 matrix using the following chromatographic workflow: activation with 50 μL acetonitrile, equilibration with 200 μL 0.1% formic acid, sample application, two times wash with 200 μL 0.1% formic acid and two times elution with 30 μL 80% acetonitrile, 0.1% formic acid. Samples were then evaporated to dryness at 40 °C in a speedvac before being resuspended in 20 μL 0.1% formic acid and stored at −20 °C until mass spectrometry analysis.

### Proteomics Mass Spectrometry Analysis

Proteomics analysis was performed on a Q-exactive HF Hybrid Quadrupole-Orbitrap Mass Spectrometer coupled with an UltiMate 3000 RSLCnano System with an EASY-Spray ion source. Two μL sample was loaded onto a C18 Acclaim PepMap 100 trap column (75 μm × 2 cm, 3 μm, 100 Å) with a flow rate of 7 μL per min, using 3% acetonitrile, 0.1% formic acid and 96.9% water as solvent. The samples were then separated on ES802 EASY-Spray PepMap RSLC C18 Column (75 μm × 25 cm, 2 μm, 100 Å) with a flow rate of 3.6 μL per minute for 40 min using a linear gradient from 1 to 32% with 95% acetonitrile, 0.1% formic acid, and 4.9% water as secondary solvent. Mass spectrometry analysis was performed using one full scan (resolution 30,000 at 200 *m*/*z*, mass range 300–1200 *m*/*z*) followed by 30 MS2 DIA scans (resolution 30,000 at 200 *m*/*z*, mass range 350–1000 *m*/*z*) with an isolation window of 10 *m*/*z*. The maximum injection times for the MS1 and MS2 were 105 and 55 ms, respectively, and the automatic gain control was set to 3·106 and 1·106, respectively. Precursor ion fragmentation was performed with high-energy collision-induced dissociation at an NCE of 26 for all samples.

The prosit intensity prediction model “Prosit_2020_intensity_hcd” was used to generate a predicted peptide library from a FASTA file of the UniProt proteome set *Synechocystis* sp. PCC 6803: UP000001425, with the sequence of *Gallionella* CbbM added.

The raw spectra were converted to mzML using MSconvert and then searched using the EncyclopeDIA v. 1.2.2. search engine. Peptides detected in at least three replicates in every sample group were tested for differential peptide abundance using the MSstats package (version 4.12.0) in R (version 4.3.1.). For every peptide in each comparison MSstats estimated fold changes and p-values adjusted for multiple hypothesis testing (Benjamini–Hochberg method) with a significance threshold of 0.01.

During data analysis, one replicate taken at a gas feed of 1% (v/v) CO2, 78% (v/v) N2, 21% (v/v) O2 was removed from further analyses as an outlier.

### Western Blot Analysis

To ensure a similar growth status of different liquid cultures, cultures were grown twice to an OD_730 nm_ of approximately 0.9 and subsequently diluted to an OD_730 nm_ of 0.2. When cultures reached an OD_730 nm_ of 0.9 for the second time, they were harvested by centrifugation (4500*g*, 4 °C, 10 min) and all remaining supernatant was removed. Cell pellets were frozen at −20 °C until further usage. For lysis, pellets were resuspended in 200 μL phosphate-buffered saline containing protease inhibitors (cOmplete, EDTA-free Protease Inhibitor Cocktail, Roche) and mixed with 50 μL of glass beads (425–600 μm diameter, Sigma-Aldrich). Homogenization was performed as described for mass spectrometry analyses. Cell lysate was separated into soluble and insoluble fractions by centrifugation (20,000*g*, 4 °C, 20 min). Protein concentrations were determined as described above using the Bio-Rad Protein Assay Dye Reagent Concentrate (Bio-Rad) and a BSA standard curve. Samples were analyzed using SDS-PAGE and Western blot. Soluble fractions were mixed 1:1 with 2× Laemmli Sample Buffer (Bio-Rad) containing 50 mM dithiothreitol. After a 5 min 95 °C denaturation step, approximately 20 μg protein of the soluble cell fraction was loaded onto precast 4–20% Mini-PROTEAN TGX Stain-Free Protein Gels (Biorad) for SDS-PAGE. SeeBlue Plus2 prestained protein standard (Invitrogen) was used as size standard. Two identically prepared gels were run at the same time, of which one was stained using QC Colloidal Coomassie Stain (Bio-Rad) according to the manufacturer’s protocol. The second gel was used for Western blotting onto a 0.2 μm PVDF membrane using Trans-Blot Turbo Mini 0.2 μm PVDF Transfer Packs (Bio-Rad) for 30 min at 25 V with an upper threshold of 1.0 A. After blocking in 5% milk powder in Tris-buffered saline containing Tween 20 (TBS-T, 20 mM Tris, pH7.5, 150 mM NaCl, 0.1% Tween 20), membranes were kept at 4 °C overnight. Subsequently, they were shaken for 30 min at room temperature and washed twice with TBS-T for 10 min. Antistrep-tag II rabbit IgG antibody (ab76949) in TBS-T (1:2000 dilution, 0.5 μg mL^–1^) was added to the membrane for 1 h at room temperature. After two 10 min washing steps using TBS-T, secondary horseradish peroxidase (HRP) conjugated goat antirabbit IgG antibody (Abcam #ab672) in TBS-T (dilution 1:5000, 0.16 μg mL^–1^) was added for 1 h at room temperature. The membrane was washed again and Clarity Western enhanced chemiluminescence (ECL) substrates (Bio-Rad) were mixed 1:1 and applied to the membrane. The membranes were then analyzed with the Odyssey FC imaging system (Li-Cor).

### Protein Expression and Purification

The DNA sequence of the plasmid used for expression is provided online (pet28_rubisco.gb). BL21­(DE3) were transformed with these pET28a-derived plasmids and for each CbbM variant, a single colony was used to inoculate 50 mL kanamycin-containing LB, which was incubated at 37 °C overnight. The next day, 200 mL kanamycin-containing 2YT in a baffled flask were inoculated using preculture to an OD_600 nm_ of 0.1 and kept shaking at 37 °C. When reaching an OD_600 nm_ of 0.6, 0.5 mM isopropyl-β-d-thiogalactopyranosid (IPTG) was added to induce protein expression and the incubation temperature was reduced to 16 °C. After 16 h, cultures were harvested by centrifugation (3005*g*, 4 °C, 20 min). Cell pellets were resuspended in 10 mL B-PER Complete Bacterial Protein Extraction (ThermoScientific) containing imidazole (20 mM) and incubated while rocking at room temperature for 25 min. The suspension was centrifuged (3005*g*, 4 °C, 20 min) and the supernatant was filtered using a 0.2 μm filter (SFCA + PF membrane, Corning). For each expressed variant, 2.5 mL Ni-NTA agarose beads (Qiagen) were prepared as described by the manufacturer. After three wash steps using B-PER containing 20 mM imidazole, the filtered supernatant was added to the beads and incubated on ice for 1 h, while rocking. The bead suspension was transferred to a 5 mL polypropylene column (Qiagen) and the flow through was collected. The column was washed three times with 5× bead volume (6.25 mL) wash buffer (50 mM Tris–HCl, 20 mM imidazole, 500 mM NaCl, pH 7.4) and the wash flow through was collected. The column was washed once with 10% elution buffer and collected. The protein was eluted in four fractions of 0.5 mL elution buffer (50 mM Tris–HCl, 300 mM imidazole, 500 mM NaCl, pH 7.4) each. The fractions for each protein variant were collected and the buffer was exchanged to a Rubisco storage buffer (20 mM MgCl_2_, 20 mM EPPS, pH 8.0) using PD10 desalting columns (Cytiva) according to the manufacturer’s instructions. Eluates were concentrated using protein concentrators (10 kDa, Cytiva) by centrifugation (3005*g*, 4 °C) until reaching a volume of approximately 500 μL. Protein concentration was measured using the Bio-Rad Protein Assay Dye Reagent Concentrate (Bio-Rad) as described above and molarity calculated using molecular weights given in Supp. Table S14. Protein samples were used for downstream assays within 24 h. Each protein purification was controlled by running SDS-PAGE as described above (compare Supp. Figure S16 for an exemplary SDS gel).

### Nano Differential Scanning Fluorimetry (nanoDSF)

The thermal stability of the Rubisco variants was determined by nano differential scanning fluorimetry (nanoDSF). The mutant variants were diluted to approximately 1 mg mL^–1^ with 100 mM EPPS and 50 mM NaHCO_3_. Three technical replicates of a blank sample (100 mM EPPS, 50 mM NaHCO_3_) and the mutant variants were transferred to Prometheus standard capillaries (NanoTemper Technologies) and placed in the Prometheus NT.48 instrument (NanoTemper Technologies). The thermal profile of the proteins was measured by the tryptophane shift and scattering at 350 and 330 nm in the temperature range from 20 to 95 °C at an increase of 1 °C min^–1^ with 23% excitation power.

### Spectrophotometric Rubisco In Vitro Assay

Spectrophotometric in vitro assays were performed, with slight modifications, according to.^8^ Rubisco variant stocks were prepared at a protein concentration of 5 μM in 50 mM NaHCO_3_ and 100 mM EPPS. Assay components (see Supp. Table S15) were mixed (except RuBP and Rubisco protein) and 80 μL were added to each used well of a UV-transparent (Corning) 96 well plate. The absorbance at 340 nm was measured on a spectrophotometer (SpectraMaxR i3x, Molecular Devices or BioTek Epoch 2 microplate reader, Agilent) every 10 to 15 s for 15 min. To respective wells, 10 μL of the respective 5 μM Rubisco stock was added, mixed carefully by pipetting and absorbance at 340 nm was measured for 15 min. In a final step, 10 μL of RuBP was added to each well, all components were added by pipetting and absorbance was measured for 15 min. The assay was performed at 30 °C in ambient air as well as in an anaerobic tent in an oxygen-free atmosphere (10% CO2, 87.9% N2, 2.1% H2). A negative control containing RuBP without RubiscO and a positive control containing 1 mM 3-phosphoglycerate with Rubico were performed. The assay was performed trice for each condition. In the anaerobic tent, all tubes containing assay reagents (assay mix, protein, substrate) were opened and let to equilibrate for a minimum of 15 min before performing the assay. Separately, standards of NADH were measured in a range from 5–625 μM and plotted to obtain a factor (β) to convert absorbance to concentration.

The obtained absorbance values were converted with the β factor (1569 L/mol) divided by two to account for two molecules of 3-phosphoglycerate produced per RuBP (*c* = (*A*/β)/2)). In GraphPad Prism, linear regression was performed on concentration per second and the slope obtained was used to calculate the rate (*k*_app_ = −(slope × 10^9^)/protein concentration). As protein concentration, we used 500 nM.

## Supplementary Material





## Data Availability

Raw sequencing data were deposited at the European Nucleotide Archive (ENA accession number PRJEB79580). The mass spectrometry proteomics data have been deposited to the ProteomeXchange Consortium via the PRIDE^66^ partner repository with the data set identifier PXD059631 (Reviewer access details: **Project accession:** PXD059631 **Token:** 5D4oc2cOEiWh). Source code used for analyses and some raw data is available on GitHub (https://github.com/ute-hoffmann/CbbM_16variants and https://github.com/ute-hoffmann/EVmut_inSilico) and Zenodo (dois: 10.5281/zenodo.14699592 and 10.5281/zenodo.13971225).

## Notes

Supplemental Notes: (1) Untargeted proteomics of CbbM-containing *Synechocystis*. (2) Estimations on CO_2_ and O_2_ concentrations in microenvironment of CbbM. Supplementary Figures, including Figures supporting mass spectrometric findings, growth data and microscopy; and supplementary Tables including more details for mass spectrometric analyses, amino acid exchange effect prediction and strain construction (PDF)
Supplementary Tables including the complete mass spectrometric data set, EVmutation predictions for CbbM and output of the sequencing analysis pipeline (XLSX)

## Abbreviations

aTc: Anhydrotetracycline
CRISPRi: CRISPR interference
DMS: Deep mutational scanning
IPTG: Isopropyl-β-d-thiogalactopyranosid
*K*_C_: Michaelis constant of Rubisco for CO_2_
*k*_cat,C_: Turnover number or catalytic constant of Rubisco for carboxylation reaction
*K*_O_: Michaelis constant of Rubisco for O_2_
RbcLS: *Synechocystis* Rubisco
RuBP: Ribulose-1,5-bisphosphate
sgRNA: Single guide RNA
*S*(%): Specificity factor of Rubisco for CO_2_ over O_2_
*Synechocystis*: *Synechocystis* sp. PCC 6803
TBS-T: Tris-buffered saline containing Tween20
*V*_C_: Limiting rate of Rubisco for CO_2_
