# Supplementary material for: A Cyanobacterial Screening Platform for Rubisco Mutant Variants
Source: ACS Synth Biol. 2025 Jul 7;14(7):2619–33. doi: 10.1021/acssynbio.5c00065 (PMC12313052; doi:10.1021/acssynbio.5c00065)
Supplement: Supplementary file 1 [file sb5c00065_si_001.pdf]

A cyanobacterial screening platform for Rubisco mutant variants

Ute A. Hoffmann[1,*], Anna Z. Schuppe[2], Axel Knave[1], Emil Sporre[1], Hjalmar Brismar[3], Elias Englund[1], Per-Olof Syrén[2] and Elton P. Hudson[1,*]

[1]Department of Protein Science, School of Engineering Sciences in Chemistry, Biotechnology and Health, Science for Life Laboratory, KTH - Royal Institute of Technology, 106 91 Stockholm, Sweden
[2]Department of Fibre and Polymer Technology, School of Engineering Sciences in Chemistry, Biotechnology and Health, Science for Life Laboratory, KTH - Royal Institute of Technology, 100 44 Stockholm, Sweden
[3]Department of Applied Physics, School of Engineering Sciences, Science for Life Laboratory, KTH - Royal Institute of Technology, 114 19 Stockholm, Sweden

*To whom correspondence should be addressed. Elton P. Hudson: Tel: +46 70 783 95 07, Email: paul.hudson@scilifelab.se; Ute A. Hoffmann: Email: ute@hoffmannk.de

Supplemental Information

Supplemental Notes

Supplemental Figures

Supplemental Tables

Supplemental References

Supplemental Note 1

Untargeted proteomics of CbbM-containing *Synechocystis*

To investigate the effect of RbcLS depletion and CbbM expression on the cells, we performed untargeted label-free DIA proteomics on the different strains when grown at 5% CO_2, 75% N_2, 20% O_2 approximately five generations after addition of aTc (Supp. Fig. S3A and B, Supp. Fig. S4A, Supp. Tables S1 and S2). We unambiguously identified 1,873 proteins. When comparing the strain *Syn*-sgRNA$_{rbc}$ *cbbM*(WT) at different gas feeds (Supp. Fig. S3C and D, Supp. Fig. S4B, Supp. Tables S3 and S4), we unambiguously detected 1,857 proteins. Both numbers correspond to a coverage of approximately 52% of annotated coding sequences.

The knockdown of RbcLS in both *Syn*-sgRNA$_{rbc}$ and *Syn*-sgRNA$_{rbc}$ *cbbM*(WT) strains showed altered abundances of carboxysome components ccmM (reduction), ccmN (increase), and ccmL (increase) compared to wild type *Synechocystis* (Supp. Fig. S3A and S3B, and Supp. Table S5). The first step in carboxysome assembly is the interaction between RbcLS, carbonic anhydrase and CcmM (1). The reduction of CcmM could be due a destabilisation of the carboxysome when its interaction partners *Synechocystis* RbcL and RbcS are reduced

We also compared the proteomic response of the *Syn*-sgRNA$_{rbc}$ *cbbM*(WT) strain at 5% CO_2 and at 1% CO_2 (Supp. FigS3C and D, Supp. Fig. S4B and Supp. Table S4). Proteomic changes of *Syn*-sgRNA$_{rbc}$ *cbbM*(WT) grown at 1% CO_2 compared to 5% CO_2, included upregulation of the bicarbonate transporter SbtA and the flavodiiron protein Flv2 (Supp. Table S4). These changes are consistent with the reported transcriptomic changes of long-term low-CO_2 acclimated wild-type *Synechocystis*, which also included an upregulation of SbtA, which belongs to the NdhR regulon, and of the *flv2–flv4* operon (2, 3).

Supplemental Note 2

Estimations of CO_2 and O_2 concentrations in microenvironment of CbbM

It is difficult to determine the cytoplasmic concentration of gaseous CO_2 and O_2 relative to the external gas feed, particularly because oxygen is produced by photosynthesis when cells are grown under photoautotrophic conditions. Furthermore, while dissolved CO_2 can permeate the cell membrane and enter the cytosol, a portion is hydrated to carbonate by the NDH-1 complex leading to cytoplasmic CO_2 concentrations which might be lower than in the culture media (1). Extracellular concentrations might help to roughly estimate the intracellular concentrations. Angermayr et al., (4) recorded a dissolved CO_2 at 145 µM in BG-11 when the gas feed was 0.5% (v/v). According to Henry's law, a gas composition of 1% CO_2 or 5% CO_2 could be expected to result in a proportional increase in dissolved CO_2 concentration to 290 µM and 1.5 mM, respectively. There are some estimates on the amount of O_2 generated by *Synechocystis* photosynthesis. Angermayr et al (4) showed that *Synechocystis* cultivated in turbidostat (OD_{730nm} = 2.5) with a gas feed of 0.5% CO_2 and 99.5% N_2 generated oxygen so that dO_2 was 75 µM; the low OD in our cultivations (OD_{720nm} = 0.2) would be expected to accumulate significantly less dO_2 (estimate 10 µM). This is significantly lower than dO_2 from air-saturated water (430 µM) and far below the estimated CO_2 concentration at both 1% as well as 5% CO_2 in the gas feed.

The K_C for CO_2 of CbbM is reported to be 276 uM (5). Therefore, in a condition with 5% CO_2 and 95% N_2, we expect the enzyme to be saturated and the carboxylation rate proportional to V_C, while at a concentration of 1% CO_2 the carboxylation rate would reflect both V_C and K_C.

A gas feed of 5% CO_2 and 20% O_2 should lead to approximately 1.5 mM dissolved CO_2 compared to air-saturated water with a dO_2 of 430 µM. Given a specificity constant for *Gallionella* Rubisco of S(%) = 10.0 ± 0.1 (5), the oxygenation reaction might play a relevant role at this condition and might determine the strain's growth rate.

Supplemental Figures

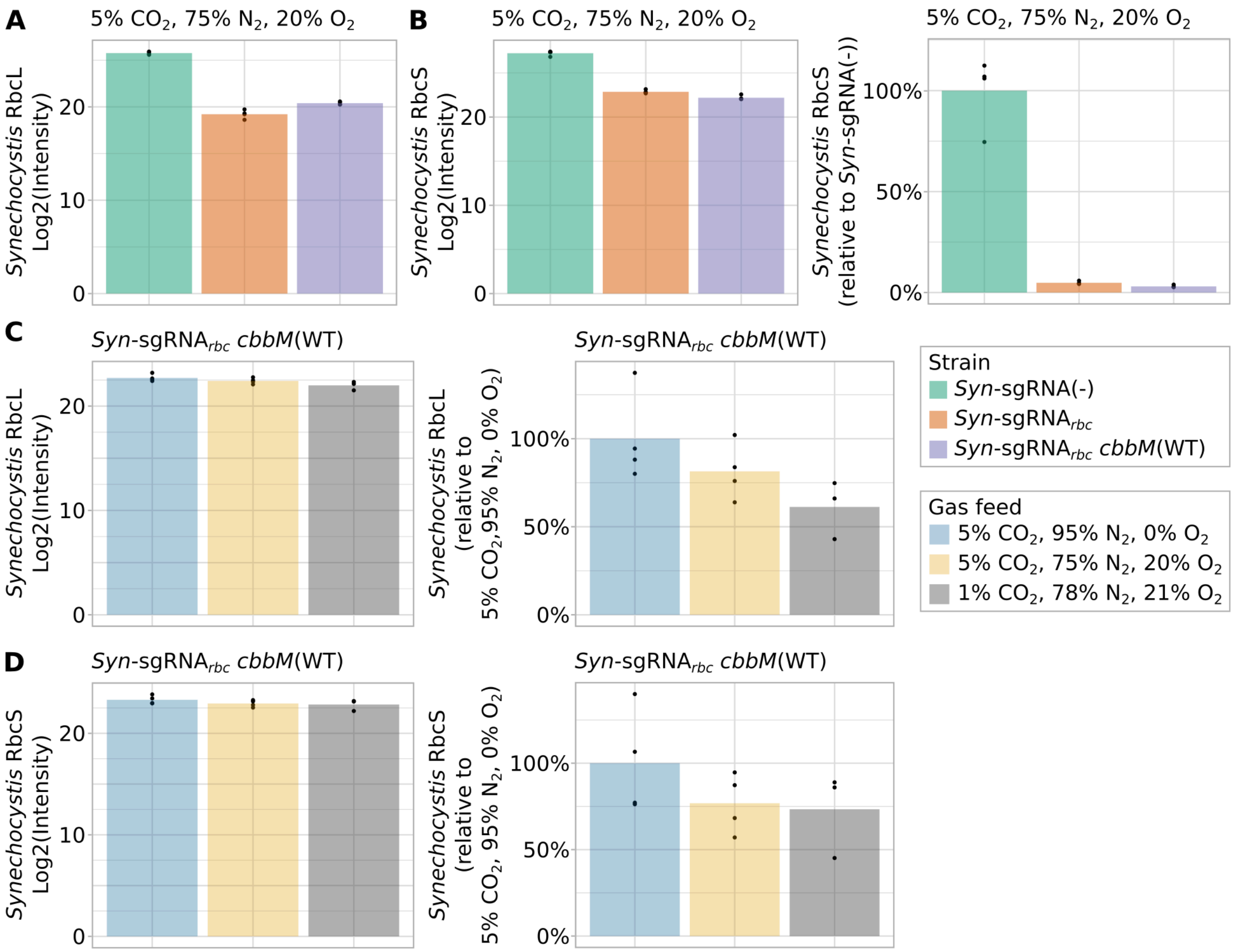

Supp. Fig. S1 (related to Fig. 1B). Mass spectrometry for *Synechocystis* Rubisco large and small subunits RbcL and RbcS. Panel (A), and left part of panels (B), (C), and (D): Log2 of measured signal intensities. Right part of panels (B), (C) and (D): relative amounts, normalized to *Syn*-sgRNA(-) (strain comparisons, panels A and B) or 5% CO_2, 95% N_2, 0% O_2 (gas feed comparisons, panels C and D). All cultivations were conducted at a light intensity of 300 µE. (**A**) Signal intensities for RbcL in different strains at 5% CO_2, 75% N_2, 20% O_2. (**B**) Data for RbcS in different strains at 5% CO_2, 75% N_2, 20% O_2. (**C**) Data for RbcL in *Syn*-sgRNA$_{rbc}$ *cbbM*(WT) at different gas feed compositions. (**D**) Data for RbcS in *Syn*-sgRNA$_{rbc}$ *cbbM*(WT) at different gas feed compositions.

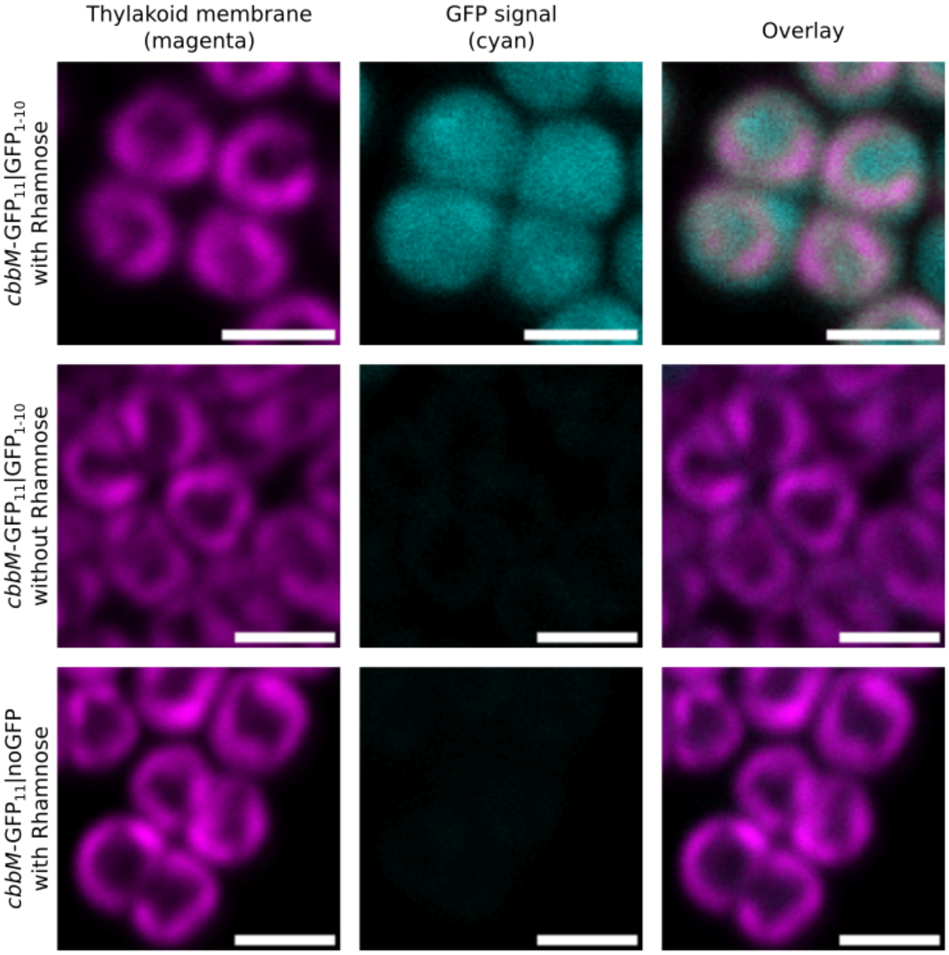

Supp. Fig. S2. Microscopy of strains expressing *Gallionella* CbbM fused to GFP11, *cbbM*-GFP_{11}|$GFP_{1\text{-}10}$ and *cbbM*-GFP_{11}|noGFP. In *cbbM*-GFP_{11}|$GFP_{1\text{-}10}$, $GFP_{1\text{-}10}$ expression is under the control of a rhamnose-inducible promoter (P_{rha}::$GFP_{1\text{-}10}$). In the control strain *cbbM*-GFP_{11}|noGFP, an antibiotic resistance cassette was introduced at the same genomic location instead of the $GFP_{1\text{-}10}$ construct. GFP fluorescence (cyan) and autofluorescence of the thylakoid membranes (magenta) is shown. All scale bars, 2 µm.

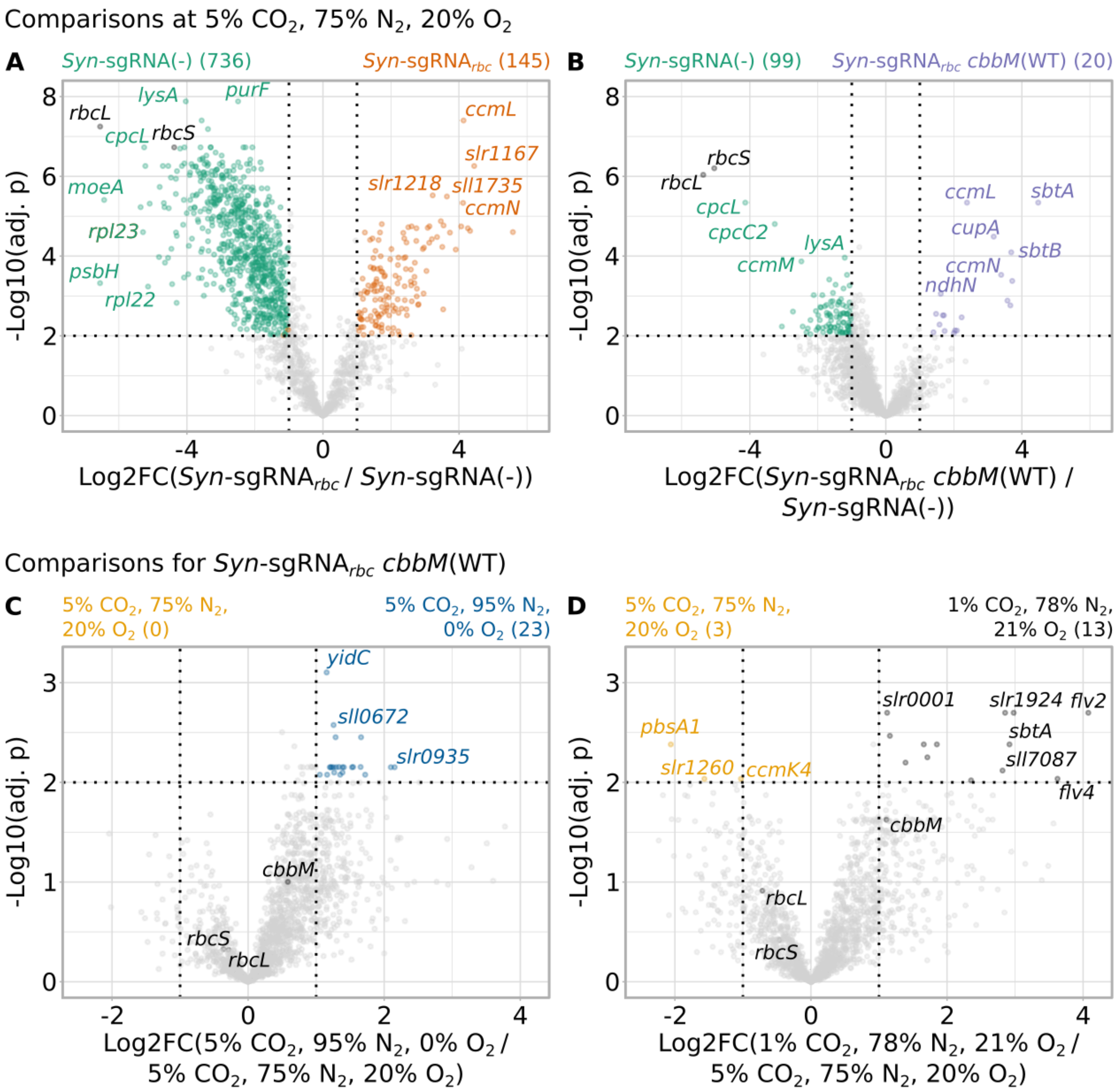

Supp. Fig. S3. Untargeted proteomics comparing the proteome of different strains at a gas feed of 5% CO_2, 75% N_2, 20% O_2 (panels **A** and **B**) and of *Syn*-sgRNA$_{rbc}$ *cbbM*(WT) at different gas feed compositions (panels **C** and **D**). Samples were taken as described for Fig. 1B and untargeted proteomics was performed. Numbers in parentheses indicate the number of proteins upregulated in the respective strain or condition with $|Log2FC| > 1$ and $adj.\ p < 0.01$ and dotted lines indicate these respective cut-off values.

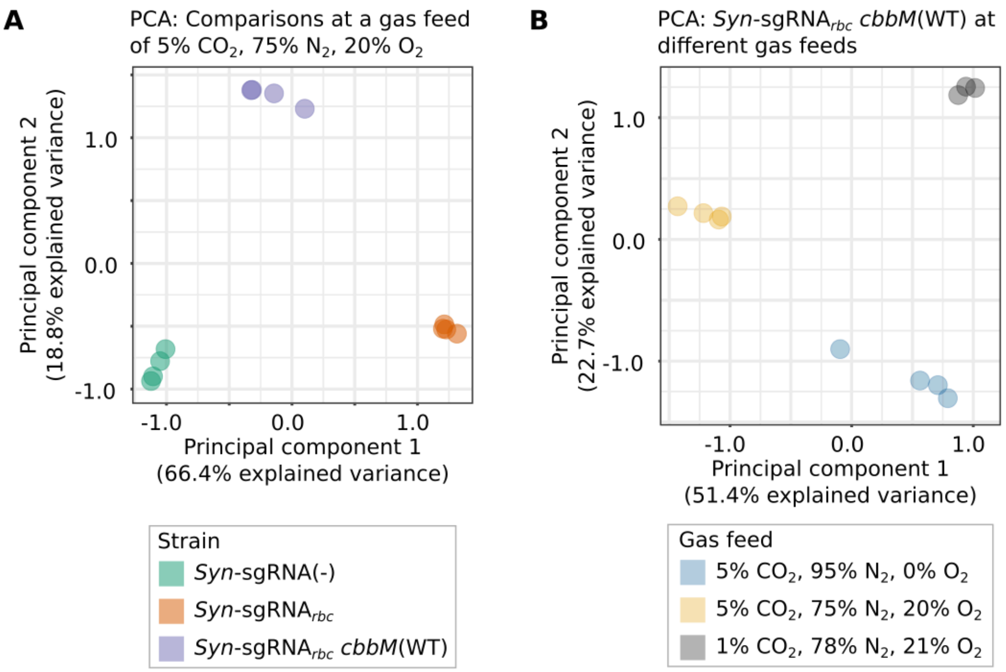

Supp. Fig. S4 (related to Supp. Fig. S3). Principal component analysis (PCA) of mass spectrometric data (Supp. Tables S1 to S4). (**A**) Comparison of different strains. (**B**) Comparison of *Syn*-sgRNA$_{rbc}$ *cbbM*(WT) at different gas feeds.

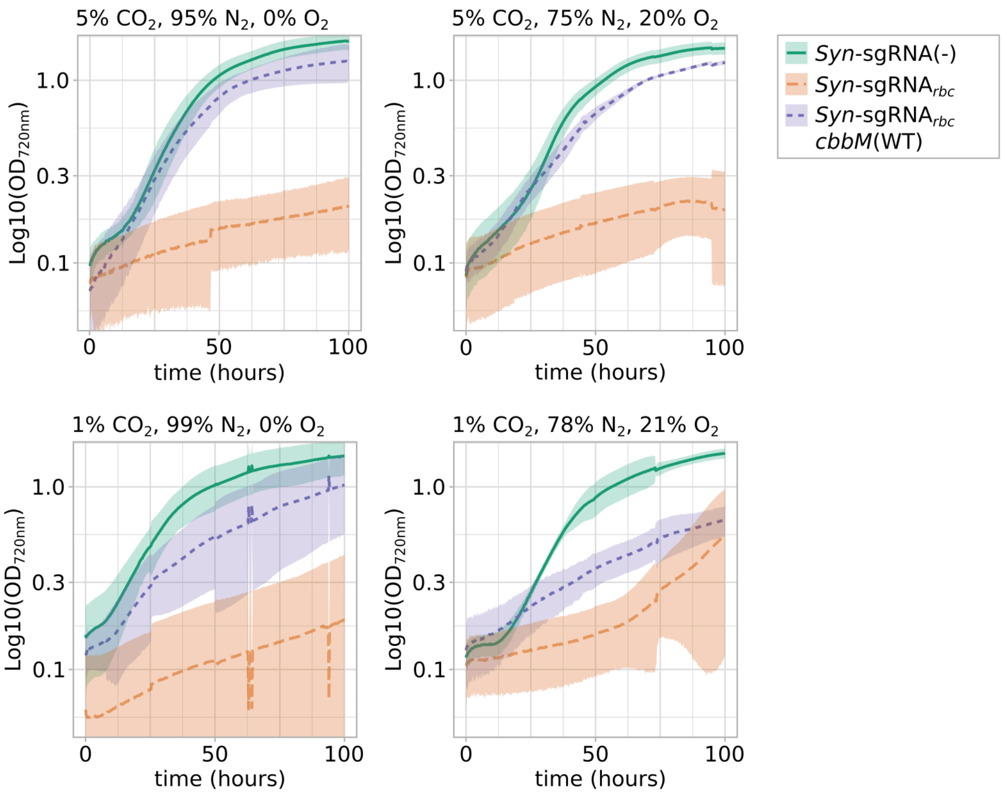

Supp. Fig. S5 (related to Fig. 1C). Growth curves of *Syn*-sgRNA(-), *Syn*-sgRNA$_{rbc}$ and *Syn*-sgRNA$_{rbc}$ *cbbM*(WT) at different gas feed CO_2/O_2 ratios after induction of the CRISPRi system using aTc (n=4, n=3 for *Syn*-sgRNA$_{rbc}$ *cbbM*(WT) at 1% CO_2, 78% N_2, 21% O_2). Growth is shown beginning from cultivating the strains at the indicated gas conditions. Light intensity was set to 300 µE. Shaded areas give the 95% confidence interval.

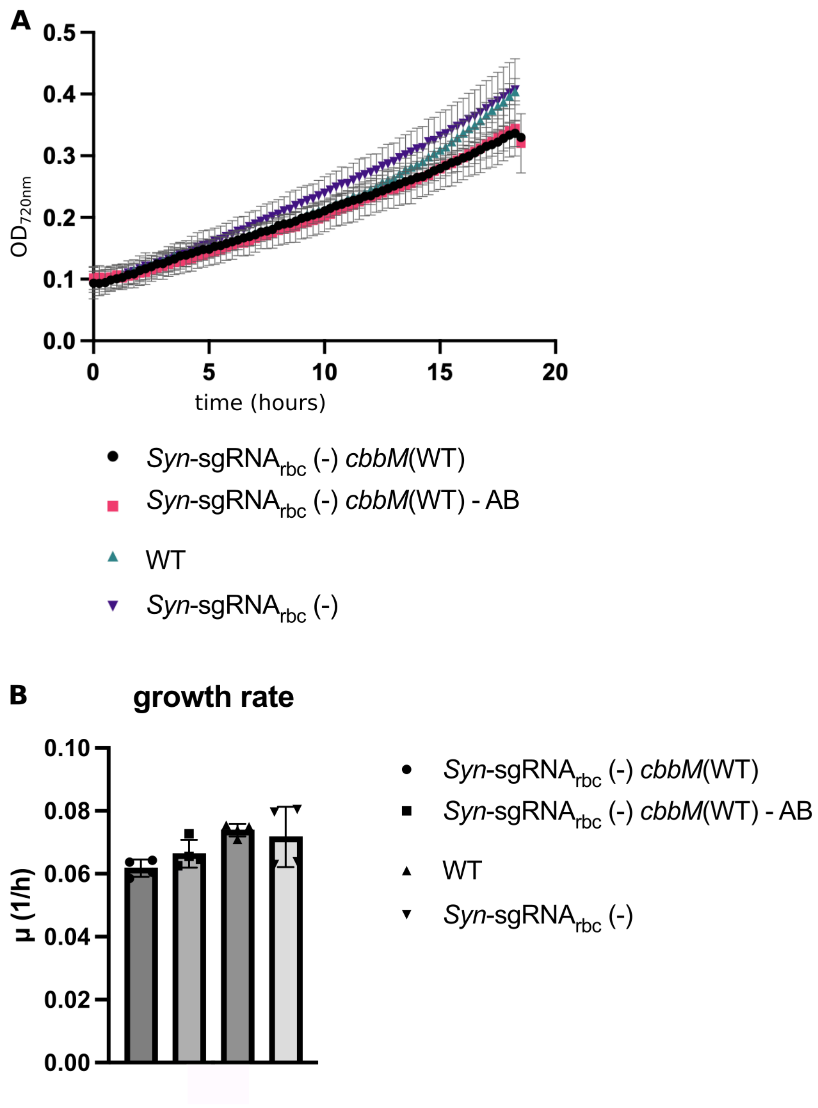

Supp. Fig. S6 (related to Fig. 1C). (**A**) Growth curve and (**B**) calculated specific growth rates of *Syn*-sgRNA(-) cbbM(WT) with and without added antibiotics (-AB), wild-type Synechocystis (WT) and *Syn*-sgRNA(-) with gas feed composition of 5% CO_2, 95% N_2 (n=4, n=3 for *Syn*-sgRNA$_{rbc}$ *cbbM*(WT)). Cell growth is shown beginning from cultivating the strains at the indicated gas conditions. Light intensity was set to 300 µE. Error bars give the standard deviation.

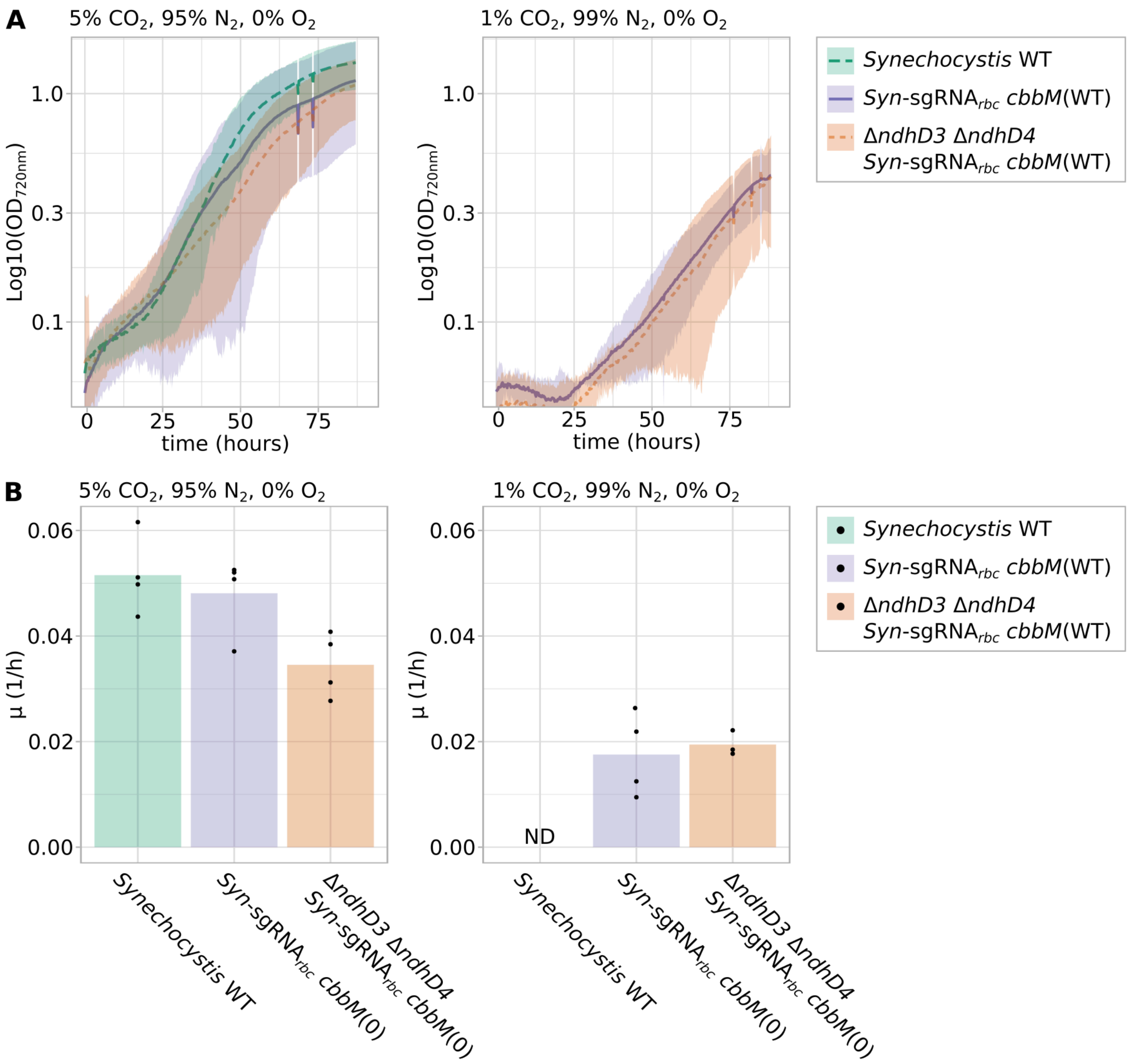

Supp. Fig. S7. Growth curves of wild-type *Synechocystis* (*Synechocystis* WT), *Syn*-sgRNA$_{rbc}$ *cbbM*(WT) and Δ*ndhD3* Δ*ndhD4* *Syn*-sgRNA$_{rbc}$ *cbbM*(WT) at different gas feed CO_2/O_2 ratios after induction of the CRISPRi system using aTc (n=4, n=3 for Δ*ndhD3* Δ*ndhD4* *Syn*-sgRNA$_{rbc}$ *cbbM*(WT) at 1% CO_2, 99% N_2, 0% O_2). Growth is shown beginning from cultivating the strains at the indicated gas conditions. Light intensity was set to 300 µE. Shaded areas give the 95% confidence interval. Growth of *Synechocystis* WT at 1% CO_2, 99% N_2, 0% O_2 was not determined (ND).

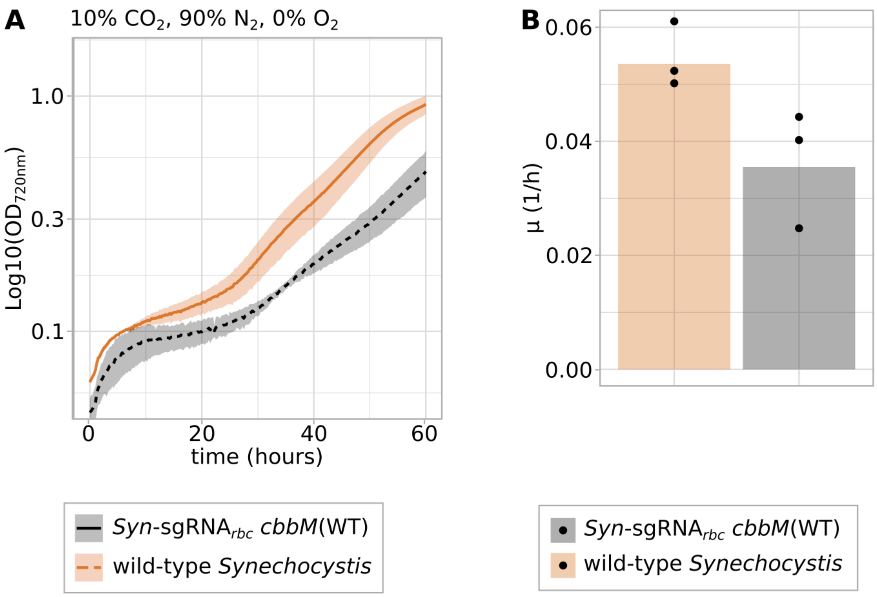

Supp. Fig. S8. Growth curves (**A**) and growth rates (**B**) of wild-type *Synechocystis* and *Syn*-sgRNA$_{rbc}$ *cbbM*(WT) at a gas feed of 10% CO_2, 90% N_2 after induction of the CRISPRi system using aTc (n=3). Growth is shown beginning from cultivating the strains at the indicated gas condition. Growth rates were determined for the time period from hours 20.5 to 40. Light intensity was set to 300 µE. Shaded areas give the standard deviation. Cultivation medium was not adjusted to reduce the effect of acidification due to the relatively high CO_2 concentration in the gas phase.

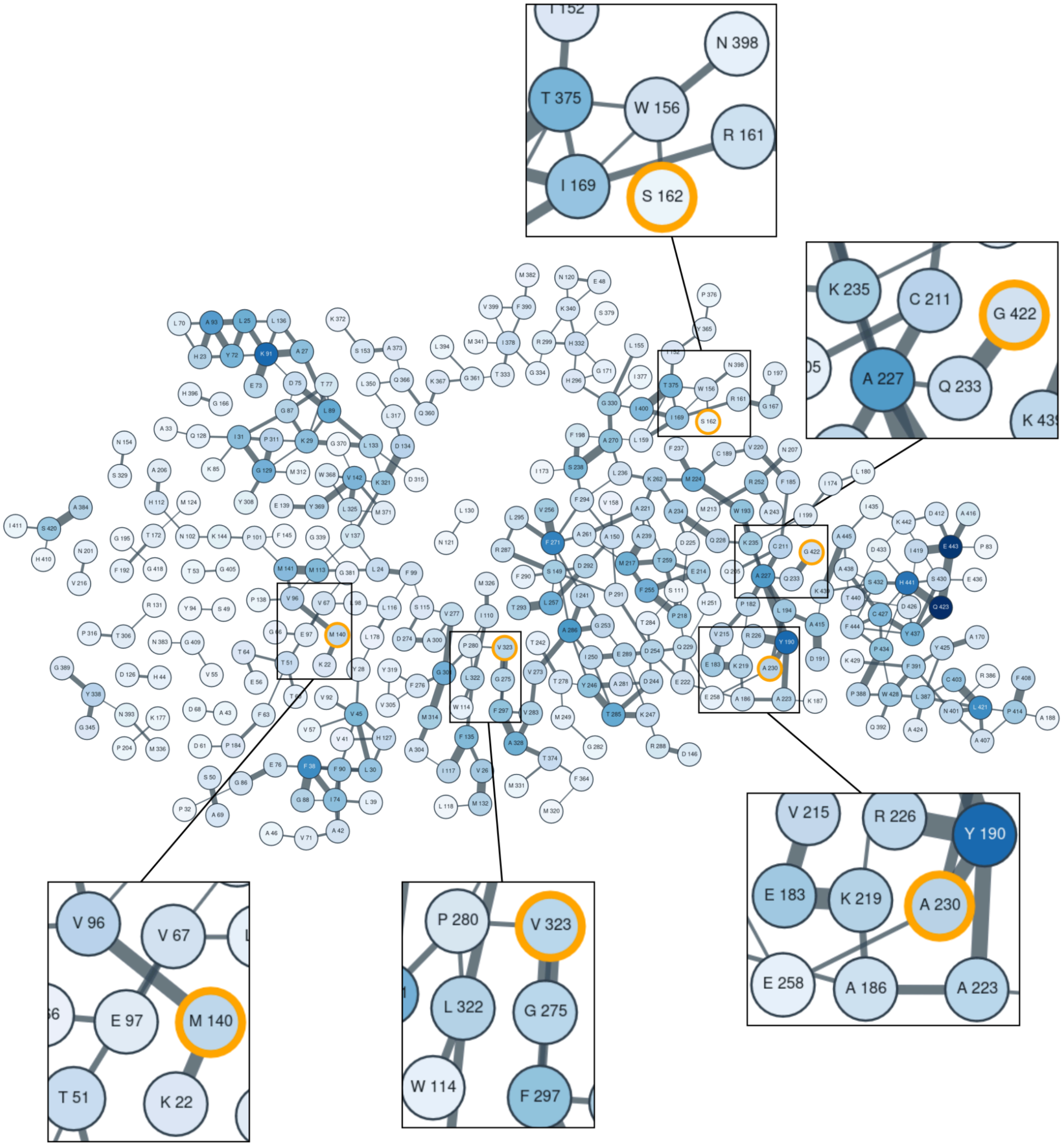

Supp. Fig. S9 (related to Fig. 2). Pair-wise couplings of amino acids in *Gallionella* Rubisco, CbbM, as given by the EVmutation web server (https://v2.evcouplings.org/). Amino acids investigated in this study are highlighted in orange. Blue color intensity and the thickness of edges indicate the degree of coupling to other residue positions. Inserts show a magnification of the immediate surroundings of the amino acid positions investigated in this study.

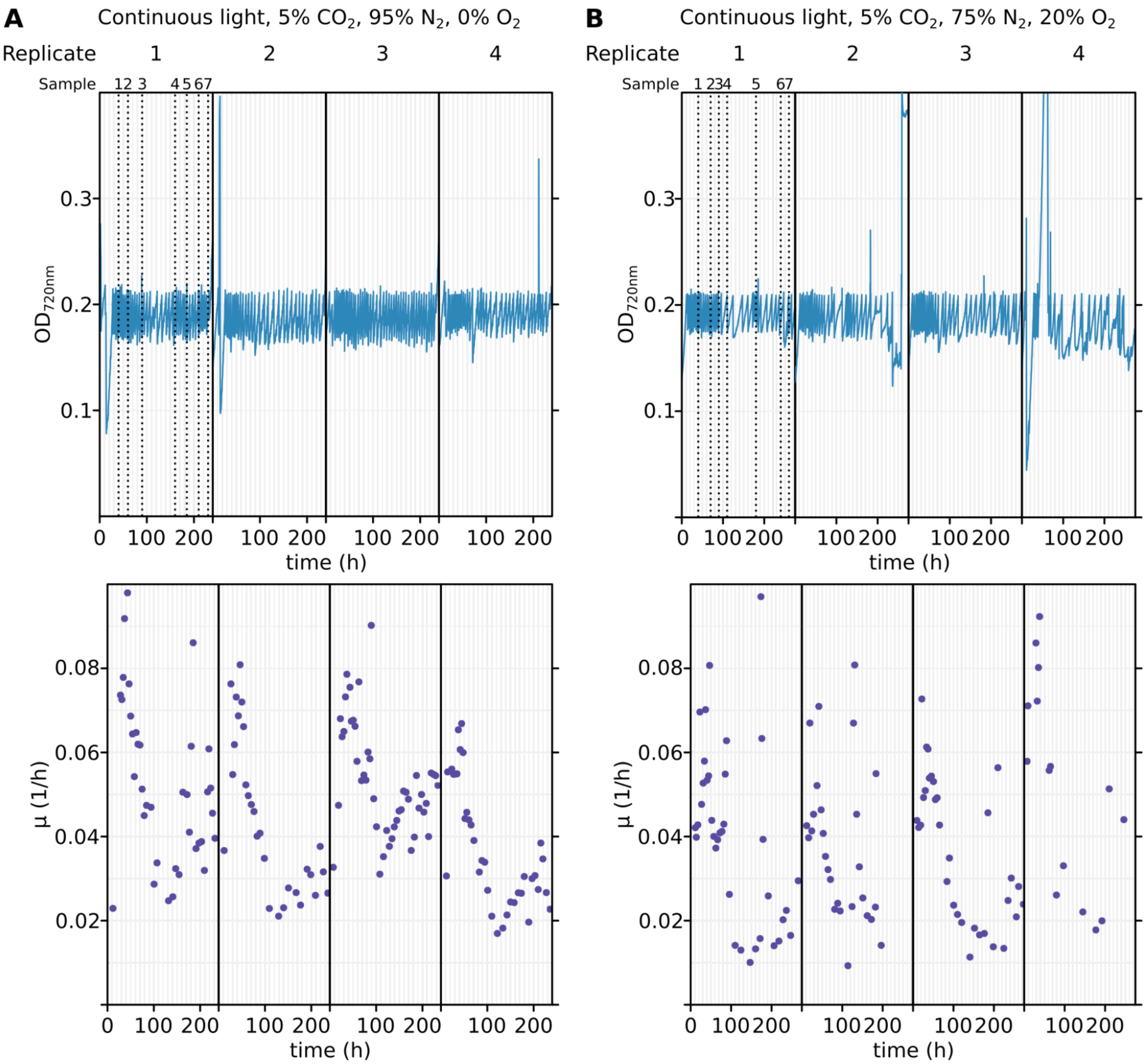

Sample	Generation (A)	Generation (B)
1	0 (+ aTc)	0 (+ aTc)
2	2.2	2.3
3	4.1	3.3
4	5.2	4.2
5	7.3	7.1
6	8.8	8.8
7	10.0	9.6

Supp. Fig. S10 (related to Fig. 3). Growth of mutant variant pool as recorded by ShinyMC. In both panels, optical density at 720 nm (OD_{720nm}) is given in the top part, and growth rates as calculated by ShinyMC in the bottom part. (**A**) Growth at continuous light of 300 µE, 5% CO_2, 95% N_2, 0% O_2. (**B**) Growth at continuous light of 300 µE, 5% CO_2, 75% N_2, 20% O_2.

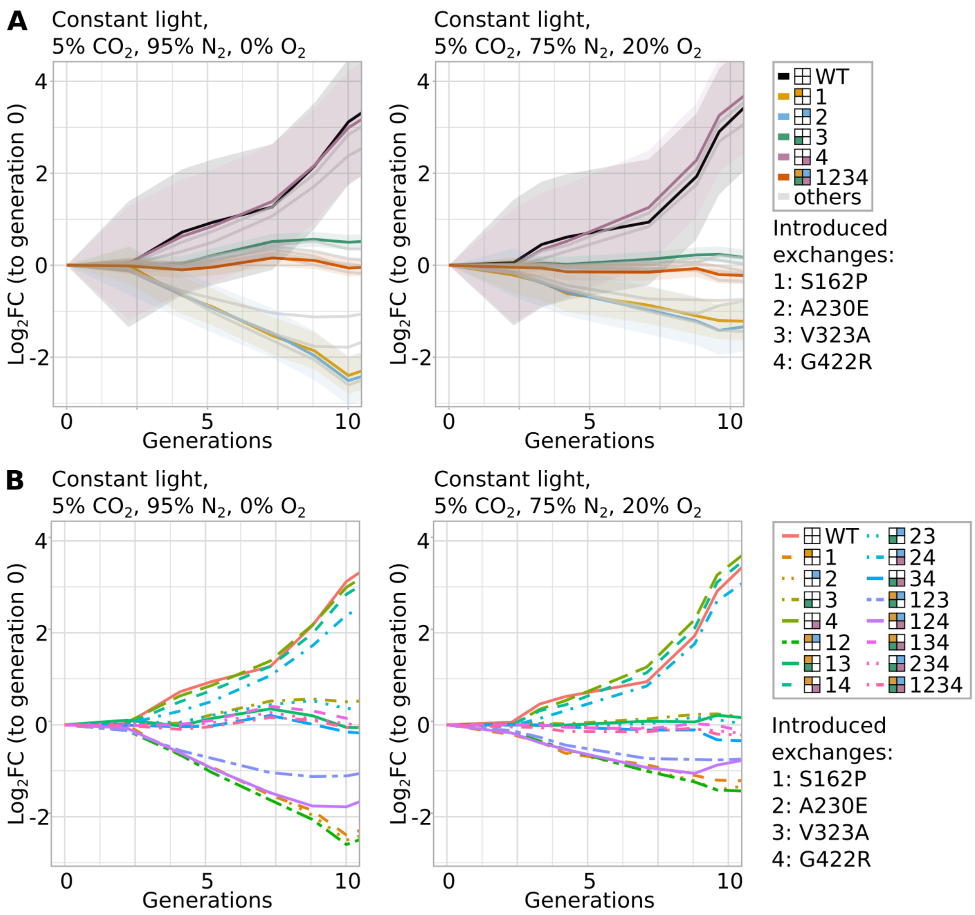

Supp. Fig. S11 (related to Fig. 3). Fitness values of competitive growth of the 16-member library at different growth conditions. All cultivations were performed at a light intensity of 300 µE. (**A**) Growth data including 95% confidence intervals as ribbons for wild-type CbbM, variants with a single amino acid exchange and of the quadruple mutant variant in color. Growth of other variants are plotted in light gray. (**B**) Fitness data of all variants present in the library. To make naming of higher-order mutants easier, different amino acid exchanges were assigned numbers based on their relative position in the protein's coding sequence and higher-order variants are called a combination of numbers: 1: S162P, 2: A230E, 3: V323A, 4: G422R. For instance, the variant containing S162P, V323A and G422R is referred to as 134.

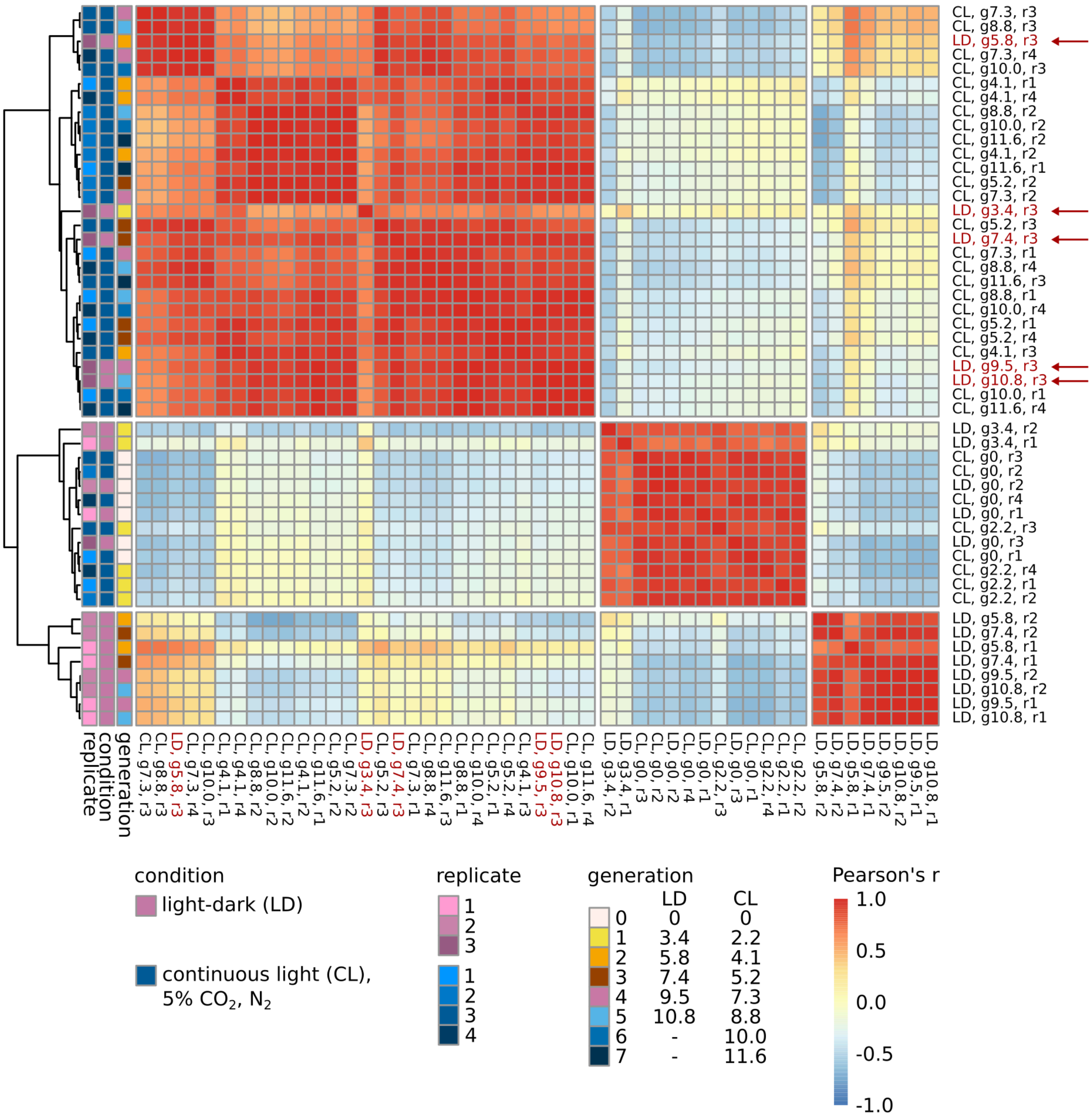

Supp. Fig. S12 (related to Fig. 3). Correlation between samples taken for cultivations with a gas feed of 5% CO_2, 95% N_2 and 0% O_2 at either continuous light (CL) or light-dark cycles (LD). Samples were clustered according to similarity. Samples for replicate number three for light-dark cycle cultivations showed a higher correlation with continuous light samples than with other light-dark replicates, probably due to stray light from the continuous light cultivation. Respective samples are indicated by red color and arrows. Since this reduced the number of usable light-dark samples to two, we decided to exclude this condition from downstream analyses.

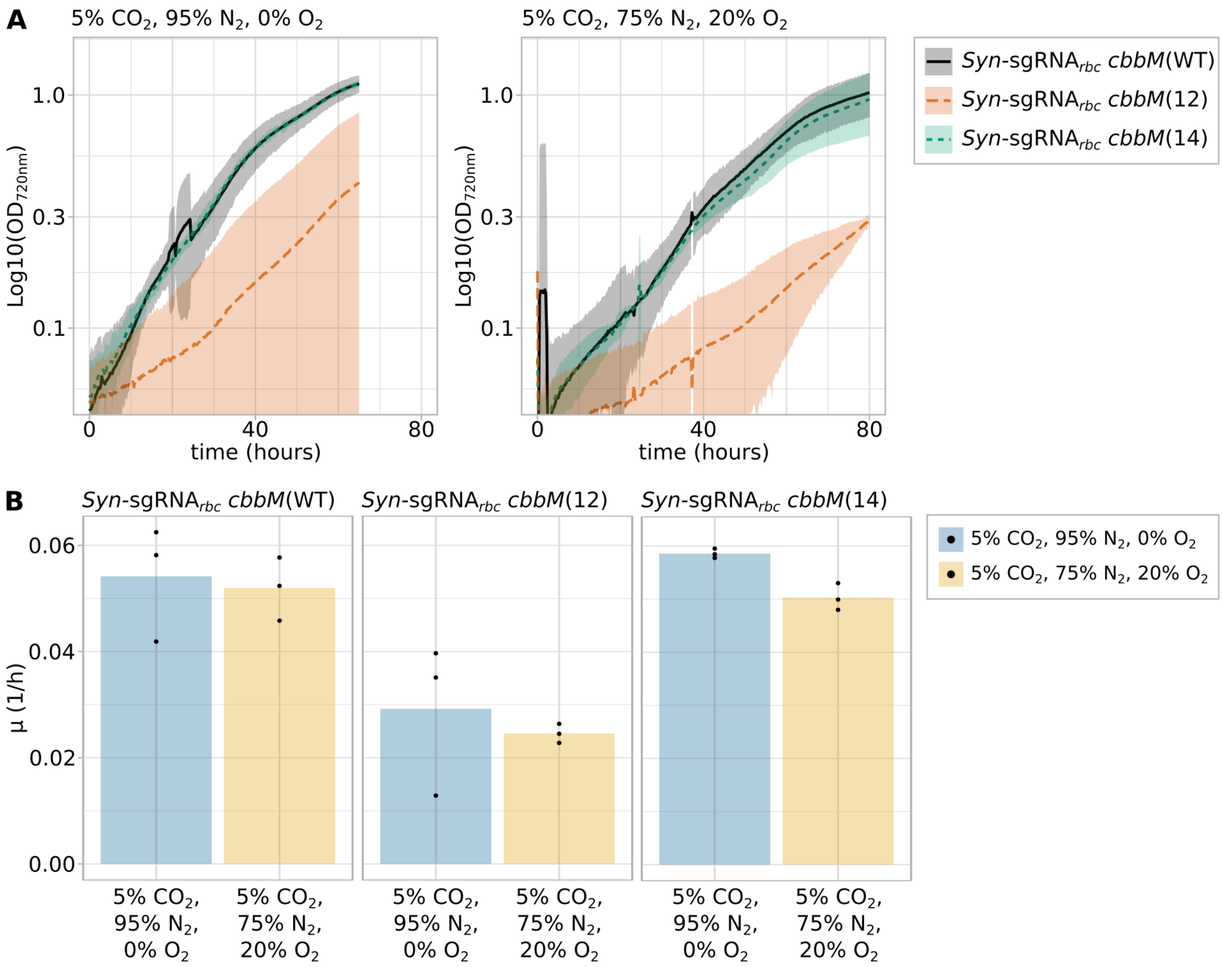

Supp. Fig. S13. Verification of pooled growth (**A**) Growth curves of *Syn*-sgRNA$_{rbc}$ *cbbM*(WT), *Syn*-sgRNA$_{rbc}$ *cbbM*(12) and *Syn*-sgRNA$_{rbc}$ *cbbM*(14) at a gas feed of 5% CO_2, 95% N_2, 0% O_2 (left) or 5% CO_2, 75% N_2, 20% O_2 (right) after induction of the CRISPRi system using aTc (n=3). Growth is shown beginning from cultivating the strains at the indicated gas conditions. Shaded areas give the 95% confidence interval. (**B**) Growth rates corresponding to growth curves shown in panel (A).

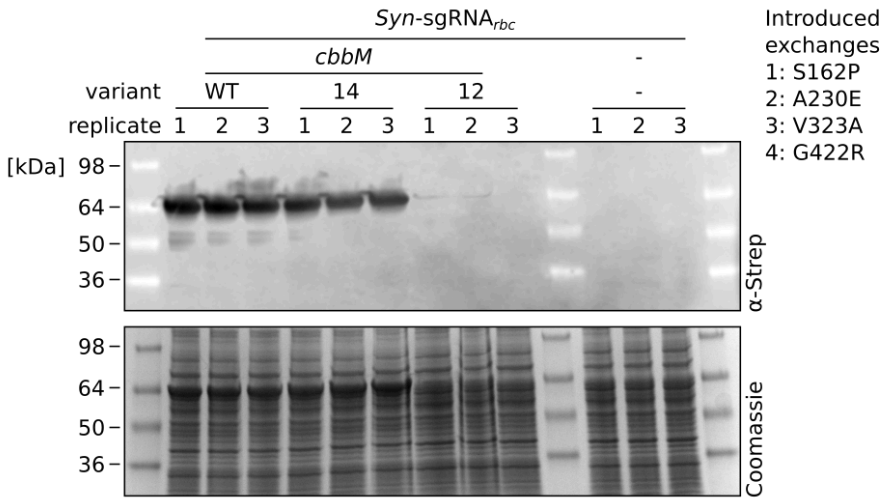

Supp. Fig. S14 (related to Fig. 4). Western blot analysis using horseradish peroxidase (HRP)-conjugated antibody directed against Strep-tag (αStrep) (top) and a Coomassie-stained SDS-PAGE gel (bottom) of the soluble cell fraction of strains expressing selected mutant variants to determine the mutant protein variants' relative abundance. Strain *Syn*-sgRNA$_{rbc}$ was used as a negative control. HRP chemiluminescence signal is overlaid with an image of the membrane. The protein of interest, N-Strep-tagged CbbM, has a size of 53 kDa. To make naming of higher-order mutants easier, different amino acid exchanges were assigned numbers based on their relative position in the protein's coding sequence and higher-order variants are called a combination of numbers: 1: S162P, 2: A230E, 3: V323A, 4: G422R. For instance, the variant containing S162P, V323A and G422R is referred to as 134.

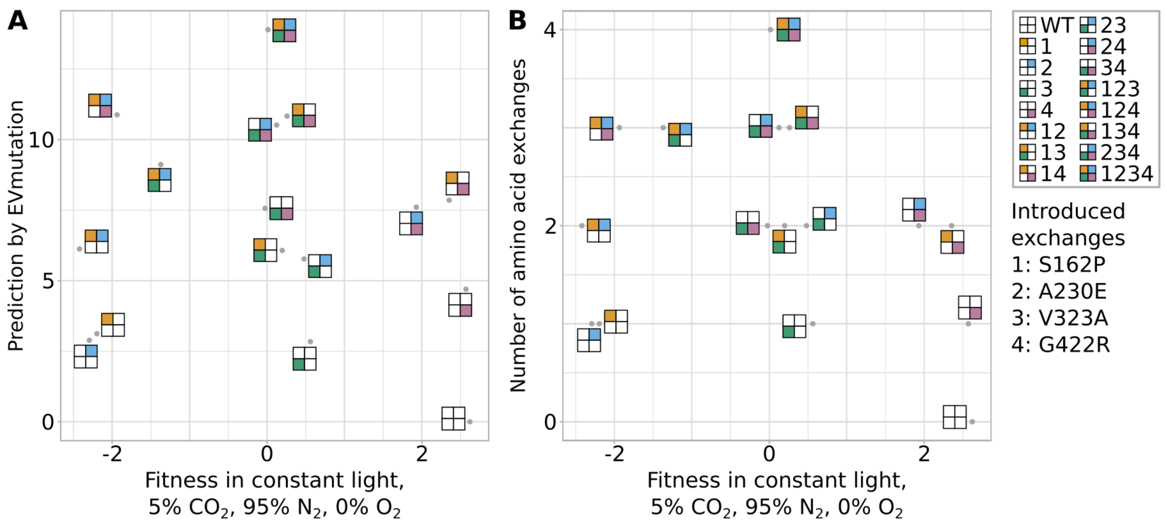

Supp. Fig. S15. (**A**) Comparison of EVmutation epistatic predictions and fitness values obtained for different variants by pooled growth at continuous light in a gas feed of 5% CO_2, 95% N_2 and 0% O_2 (compare Fig. 4). (**B**) Comparison of the number of introduced amino acid exchanges and fitness values obtained for different variants by pooled growth at continuous light in a gas feed of 5% CO_2, 95% N_2 and 0% O_2 (compare Fig. 4). For both panels, comparisons to the continuous light cultivation at 5% CO_2, 75% N_2, 20% O_2 are not shown since fitness values for the respective cultivations were highly correlated with the results for the cultivation using an oxygen-free gas feed. For reasons of clarity, we depict every variant as consisting of four boxes which represent the four different introduced amino acid exchanges. If a box is colored, the exchange was introduced and if left white, the exchange is absent. Furthermore, to make naming of higher-order mutants easier, different amino acid exchanges were assigned numbers based on their relative position in the protein's coding sequence and higher-order variants are called a combination of numbers: 1: S162P, 2: A230E, 3: V323A, 4: G422R. For instance, the variant containing S162P, V323A and G422R is referred to as 134.

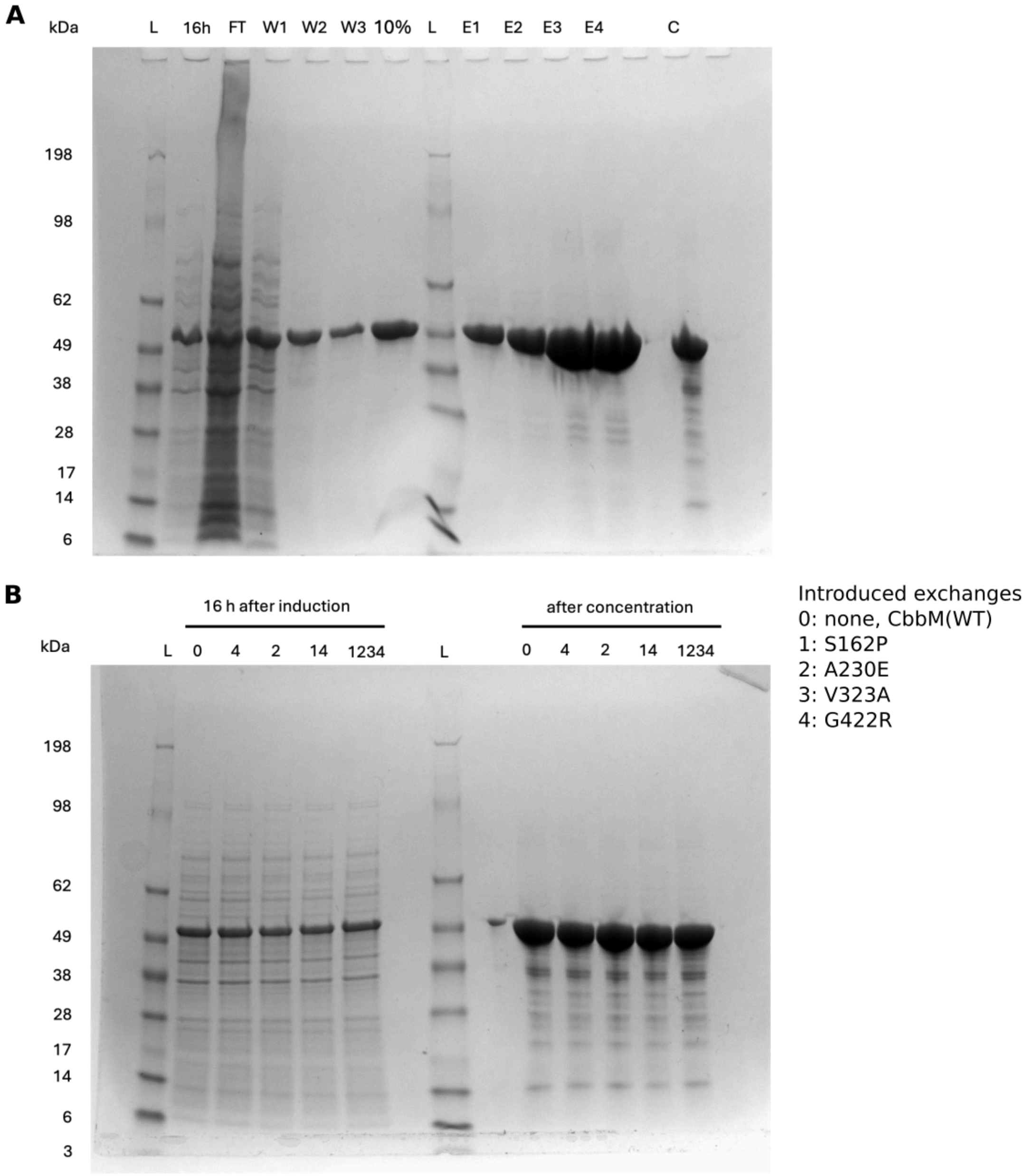

Supp. Fig. S16 (related to Fig. 4C and 4D). SDS-PAGE analysis of (**A**) one exemplary protein purification for CbbM(WT) and (**B**) protein lysates and concentrates of all purified protein variants. Gels were stained with Coomassie. Abbreviations are L: ladder, 16h: protein lysate 16 h post induction, FT: flow-through, W1 to W3: washing steps 1 to 3, 10%: washing step with 10% elution buffer, E1 to E4: eluate 1 to 4, C: concentrated eluate. Introduced amino acid exchanges are encoded as 0: none, 1: S162P, 2: A230E, 3: V323A, 4: G422R. For higher-order

variants, these numbers were combined. For instance CbbM(14) combines exchanges S162P and G422R.

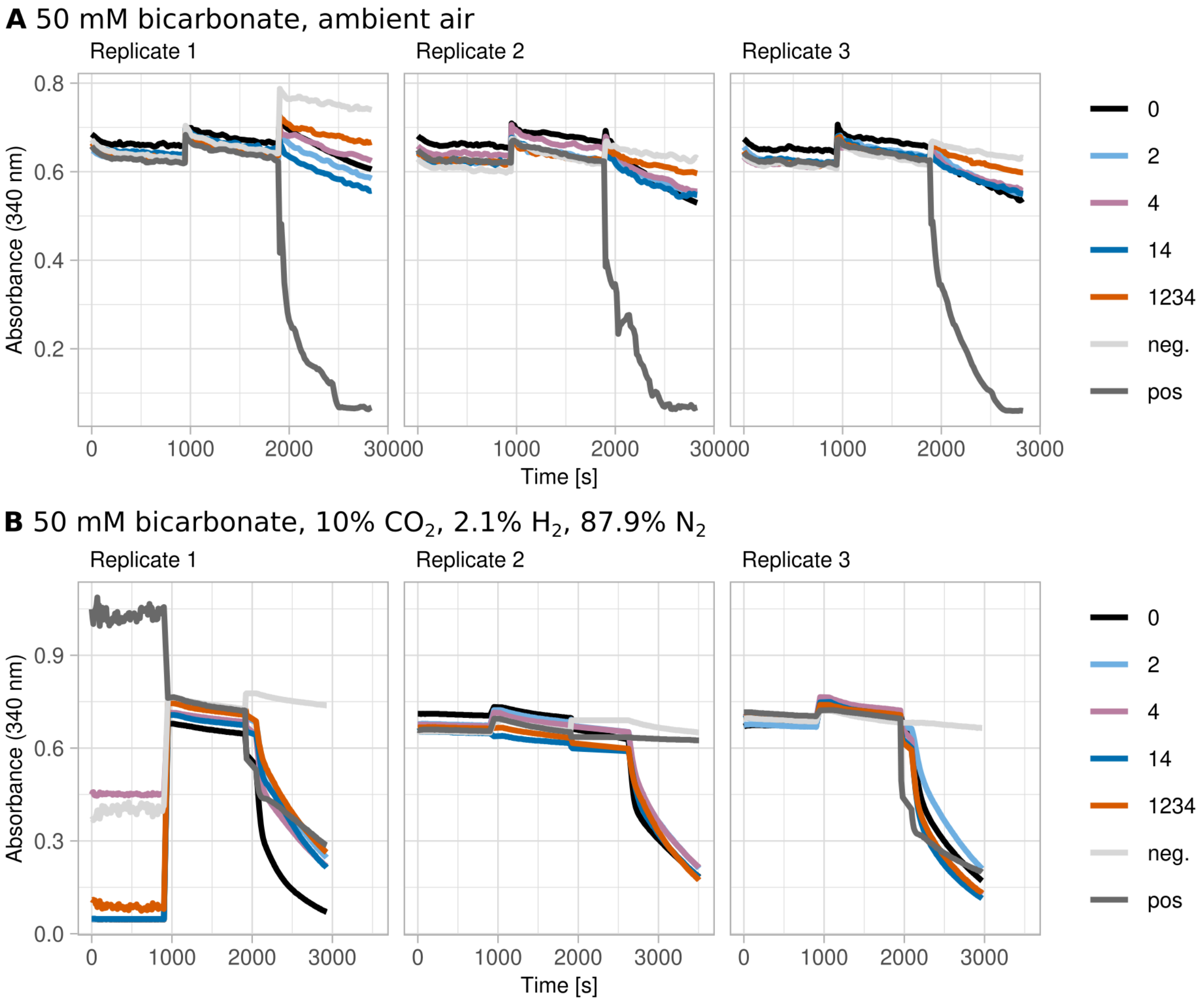

Supp. Fig. S17 (related to Fig. 4D). Spectroscopic data of the *in vitro* assays of the three measured replicates in (**A**) an oxygen-containing or (**B**) an oxygen-free atmosphere. The assays were conducted either in ambient air (panel A, approx. 0.04% CO_2, 78% N_2, 21% O_2) or in an oxygen-free gas atmosphere (panel B, 10% CO_2, 2.1% H_2, 87.9% N_2, 0% O_2). In both cases, 50 mM bicarbonate was added to the reaction mixture.

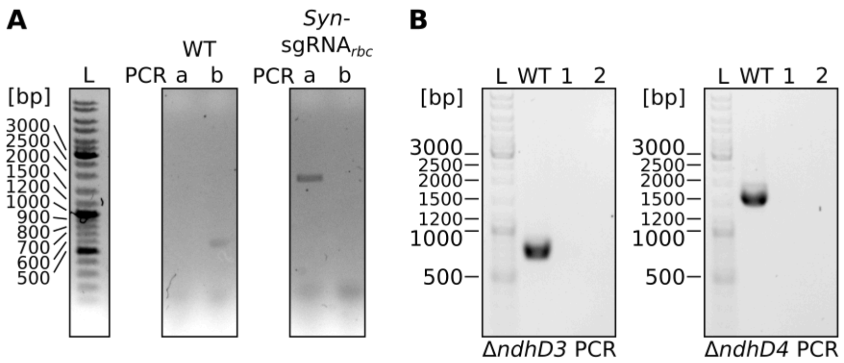

Supp. Fig. S18. Agarose TAE gels with products of PCR performed with primers to verify full segregation of strains used for further strain engineering and experiments. (**A**) PCRs run with primer pairs a) P01/P02, which gives no PCR product when the wild-type locus is present and a product of 1.6 kilobase pairs upon integration of the CRISPRi construct, and b) P03/P04, which gives a product of 528 base pairs with the wild-type locus and no band upon integration of the CRISPRi construct, on wild-type Synechocystis (WT) and *Syn*-sgRNA$_{rbc}$. (**B**) PCRs run with primer pairs P05/P06 (Δ*ndhD3* PCR) and P07/P08 (Δ*ndhD4* PCR) on WT and two clones of Δ*ndhD3* Δ*ndhD4* *Syn*-sgRNA$_{rbc}$ (numbered 1 and 2). Both primer pair combinations give no product if the respective gene was deleted and products of 738 base pairs (Δ*ndhD3* PCR) and approximately 1.6 kilobase pairs (Δ*ndhD4* PCR) when using the wild-type locus.

Supplemental Tables

For supplemental Tables S1 to 8, S11 to S14 and S16, see separate Excel workbook SuppTables_Synechocystis_CbbM-screening.xlsx. Tables are presented as an Excel workbook since they would exceed the limitation of a pdf file.

Supplementary Tables as part of separate Excel file:

- Supp. Table S1: Comparison of strains *Syn*-sgRNA(-) and *Syn*-sgRNA$_{rbc}$ at a gas feed of 5% CO_2, 75% N_2, 20% O_2 using mass spectrometry. Higher log2FC values indicate that a protein is more abundant in *Syn*-sgRNA$_{rbc}$. Column “Entry” gives the UniProtKB primary accession number. Columns “Locus tags”, “Gene names” and “Protein names” were computationally added based on these accession numbers and are not necessarily complete.
- Supp. Table S2: Comparison of strains *Syn*-sgRNA(-) and *Syn*-sgRNA$_{rbc}$ *cbbM*(WT) at a gas feed of 5% CO_2, 75% N_2, 20% O_2 using mass spectrometry. Higher log2FC values indicate that a protein is more abundant in *Syn*-sgRNA$_{rbc}$ *cbbM*(WT). Column “Entry” gives the UniProtKB primary accession number. Columns “Locus tags”, “Gene names” and “Protein names” were computationally added based on these accession numbers and are not necessarily complete.
- Supp. Table S3: Comparison of strain *Syn*-sgRNA$_{rbc}$ *cbbM*(WT) at gas feeds of 5% CO_2, 75% N_2, 20% O_2 and 5% CO_2, 95% N_2, 0% O_2 using mass spectrometry. Higher log2FC values indicate that a protein is more abundant in 5% CO_2, 95% N_2, 0% O_2. Column “Entry” gives the UniProtKB primary accession number. Columns “Locus tags”, “Gene names” and “Protein names” were computationally added based on these accession numbers and are not necessarily complete.
- Supp. Table S4: Comparison of strain *Syn*-sgRNA$_{rbc}$ *cbbM*(WT) at gas feeds of 5% CO_2, 75% N_2, 20% O_2 and 1% CO_2, 78% N_2, 21% O_2 using mass spectrometry. Higher log2FC

values indicate that a protein is more abundant in 1% CO_2, 78% N_2, 21% O_2. Column "Entry" gives the UniProtKB primary accession number. Columns "Locus tags", "Gene names" and "Protein names" were computationally added based on these accession numbers and are not necessarily complete.

Supp. Tables S5, S6 and S7 are part of this pdf file, see below.

- Supp. Table S8: EVmutation predictions for wild-type CbbM, output from web server https://v2.evcouplings.org/ and downloaded on 13th May 2022.

Supp. Table S9 is part of this pdf file, see below.

- Supp. Table S10: Output of nf-core-crispripipeline giving the fitness values of different CbbM mutant variant strains at different conditions (HL_5N2: continuous light, gas feed 5% CO_2, 95% N_2, 0% O_2; HL_5O2: continuous light, gas feed 5% CO_2, 75% N_2, 20% O_2, pool: pool of all strains before inoculation of photobioreactors). For different time points (measured in generations), DESeq2 output is given (baseMean, log2FoldChange, lfcSE).

Supp. Tables S11, S12, S13, S14 and S15 are part of this pdf file, see below.

Supp. Table S5. Subset of Supplementary Tables S1 and S2 with data for the Rubisco operon, carboxysome, carbonic anhydrase, CmpR and NdhR regulon according to (1). Comparisons are given for different strains at a gas feed of 5% CO_2, 75% N_2, 20% O_2 (compare Supp. Fig. S3 and S4). "ND" signifies that the respective number could not be determined, e.g because the protein of interest was not detected in one of the samples. Log2 fold changes (Log2FC) above 1.0 or below -1.0 with associated significant adjusted p values (*adj. p < 0.01*) were marked in green and red, respectively.

			Syn-sgRNA$_{rbc}$/ *Syn*-sgRNA(-)		*Syn*-sgRNA$_{rbc}$ *cbbM*(WT)/ *Syn*-sgRNA(-)	
	Locus tag	Gene name	Log2FC	adj. p	Log2FC	adj. p
Rubisco	*slr0009*	*rbcL*	**-6.55**	5.7E-8	**-5.37**	9.1E-7
Rubisco	*slr0012*	*rbcS*	**-4.37**	1.9E-7	**-5.04**	6.3E-7
Carboxysome	*sll1028*	*ccmK2*	**3.47**	1.3E-5	0.78	1.2E-1
Carboxysome	*sll1029*	*ccmK1*	0.70	4.1E-1	0.49	6.7E-1
Carboxysome	*sll1030*	*ccmL*	**4.13**	4.0E-8	**2.39**	4.6E-6
Carboxysome	*sll1031*	*ccmM*	**-2.64**	5.1E-6	**-2.48**	1.4E-4
Carboxysome	*sll1032*	*ccmN*	**4.12**	4.6E-6	**3.39**	3.0E-4
Carboxysome	*slr0169*	*ccmP*	**-3.40**	4.4E-6	-0.35	3.7E-1
Carboxysome	*slr0436*	*ccmO*	0.59	1.0E-1	1.05	3.6E-2
Carboxysome	*slr1838*	*ccmK3*	-0.04	9.0E-1	-0.60	1.1E-1
Carboxysome	*slr1839*	*ccmK4*	**-1.90**	2.3E-5	-0.56	6.8E-2
Carbonic anhydrase	*slr1347*	*ccaA*	-1.18	3.0E-2	-1.59	2.7E-2
CmpR regulon	*slr0042*	*slr0042*	-1.11	3.0E-2	0.13	8.5E-1
CmpR regulon	*slr0043*	*cmpC*	ND	ND	-0.55	2.0E-1
NdhR regulon	*sll1594*	*ndhR*	-0.01	9.8E-1	0.85	4.1E-2
NdhR regulon	*slr2011*	*slr2011*	1.02	2.0E-2	0.21	6.8E-1

NdhR regulon	*sll1733*	*ndhD3*	ND	ND	**3.66**	1.7E-3
NdhR regulon	*sll1734*	*cupA*	**1.70**	1.0E-4	**3.17**	3.2E-5
NdhR regulon	*sll1735*	*sll1735*	**3.64**	3.2E-6	**1.71**	3.1E-3
NdhR regulon	*slr1512*	*sbtA*	0.80	1.2E-2	**4.48**	4.6E-6
NdhR regulon	*slr1513*	*sbtB*	**2.22**	1.1E-4	**3.69**	8.0E-5
NdhR regulon	*sll0529*	*sll0529*	**-2.76**	7.4E-7	-0.13	6.0E-1

Supp. Table S6. Subset of Supplementary Tables S3 and S4 with data for the Rubisco operon, carboxysome, carbonic anhydrase, CmpR and NdhR regulon according to (1). Comparisons are given for strain *Syn*-sgRNA$_{rbc}$ *cbbM*(WT) at different gas feeds (compare Supp. Fig. S3 and S4). Log2 fold changes (Log2FC) above 1.0 or below -1.0 with associated significant adjusted p values (adj. p < 0.01) were marked in green and red, respectively.

			5% CO_2, 95% N_2, 0% O_2 / 5% CO_2, 75% N_2, 20% O_2		1% CO_2, 78% N_2, 21% O_2 / 5% CO_2, 75% N_2, 20% O_2	
	Locus tag	Gene name	Log2FC	adj. p	Log2FC	adj. p
Rubisco	*slr0009*	*rbcL*	-0.28	4.8E-1	-0.71	1.2E-1
Rubisco	*slr0012*	*rbcS*	-0.36	4.7E-1	-0.46	3.8E-1
Carboxysome	*sll1028*	*ccmK2*	0.34	3.9E-1	1.25	2.6E-2
Carboxysome	*sll1029*	*ccmK1*	1.03	2.4E-1	2.41	3.4E-2
Carboxysome	*sll1030*	*ccmL*	-0.42	2.7E-1	0.42	3.1E-1
Carboxysome	*sll1031*	*ccmM*	0.59	2.3E-1	0.07	9.1E-1
Carboxysome	*sll1032*	*ccmN*	0.01	9.8E-1	0.16	8.4E-1
Carboxysome	*slr0169*	*ccmP*	0.11	9.0E-1	-0.33	7.1E-1
Carboxysome	*slr0436*	*ccmO*	0.19	7.2E-1	0.33	5.5E-1
Carboxysome	*slr1838*	*ccmK3*	0.27	6.0E-1	-0.61	2.7E-1
Carboxysome	*slr1839*	*ccmK4*	0.57	3.3E-2	**-1.03**	9.2E-3
Carbonic anhydrase	*slr1347*	*ccaA*	1.07	1.3E-1	0.98	2.0E-1
CmpR regulon	*slr0040*	*cmpA*	0.92	5.4E-1	-1.15	4.7E-1
CmpR regulon	*slr0042*	*slr0042*	0.47	6.2E-1	-1.60	1.3E-1
CmpR regulon	*slr0043*	*cmpC*	0.21	5.8E-1	-1.45	1.7E-2
NdhR operon	*sll1594*	*ndhR*	-0.09	8.4E-1	1.13	3.2E-2
NdhR operon	*sll1733*	*ndhD4*	-1.03	2.1E-1	-0.82	3.5E-1
NdhR operon	*sll1734*	*cupA*	-0.59	4.5E-1	0.29	7.3E-1

NdhR operon	*sll1735*	*sll1735*	-0.41	5.0E-1	0.84	2.0E-1
NdhR operon	*slr1512*	*sbtA*	0.43	3.8E-1	**2.92**	4.2E-3
NdhR operon	*slr1513*	*sbtB*	-1.16	5.5E-2	2.07	1.6E-2
NdhR operon	*sll0529*	*sll0529*	0.27	2.7E-1	1.12	1.1E-2

Supp. Table S7. Proteins with |Log2FC| > 1 and p.adj < 0.01 in comparison of gas feeds of 5% CO_2, 95% N_2, 0% O_2 and 5% CO_2, 75% N_2, 20% O_2 for *Syn*-sgRNA$_{rbc}$ *cbbM*(WT). Subset of Supp. Table S3 showing only the significantly changed proteins.

Locus tag	Gene name	Gene product	Log2FC	adj. p
slr0935	*slr0935*	hypothetical protein	2.2	0.007
sll0565	*sll0565*	hypothetical protein	2.1	0.007
slr1261	*slr1261*	hypothetical protein	1.7	0.008
sll0002	*ponA*	penicillin-binding protein, PBP1	1.7	0.004
sll1925	*sll1925*	hypothetical protein	1.7	0.007
sll0813	*ctaC*	cytochrome *c* oxidase subunit II	1.5	0.007
slr1835	*psaB*	P700 apoprotein subunit Ib	1.5	0.007
sll1682	*sll1682*	alanine dehydrogenase	1.4	0.007
sll0226	*ycf4*	photosystem I assembly related protein	1.4	0.008
sll1019	*gloB*	hydroxyacylglutathione hydrolase	1.4	0.007
slr1470	*slr1470*	PSII auxiliary membrane protein	1.4	0.008
sll1317	*petA*	apocytochrome *f*; component of cytochrome b_6f complex	1.3	0.007
sll1316	*petC2*	cytochrome b_6f complex iron-sulfur subunit 2	1.3	0.007
sll1656	*sll1656*	hypothetical protein	1.3	0.004
slr0369	*slr0369*	RND multidrug efflux transporter	1.3	0.008
sll0672	*pacL*	cation-transporting p-type ATPase PacL	1.3	0.003
sll1453	*nrtD*	nitrate/nitrite transport system ATP-binding protein	1.3	0.007
slr1790	*slr1790*	hypothetical protein	1.2	0.007
slr0906	*psbB*	photosystem II core light harvesting protein	1.2	0.007
slr1755	*gpsA*	NAD+ dependent glycerol-3-phosphate dehydrogenase	1.2	0.007
slr0657	*lysC*	aspartate kinase	1.2	0.008
slr1471	*yidC*	Membrane protein insertase YidC	1.2	0.001

sll0851	*psbC*	photosystem II CP43 protein	1.1	0.008

Supplementary Table S8 is part of the Excel workbook SuppTables_Synechocystis_CbbM-screening.xlsx

Supp. Table S9. EVmutation epistatic predictions for all 16 variants included in the mutant pool used for screening. WT: wild-type CbbM, 1: S162P, 2: A230E, 3: V323A, 4: G422R. Variants are sorted according to the EVmutation epistatic score. This score is calculated based on the residue conservation at a specific site and its couplings to other residue positions.

Mutant name	Amino acid exchanges	EVmutation epistatic score
WT	none	0.0
3	V323A	2.8
2	A230E	2.9
1	S162P	3.1
4	G422R	4.7
23	A230E, V323A	5.8
13	S162P, V323A	6.1
12	S162P, A230E	6.1
34	V323A, G422R	7.6
24	A230E, G422R	7.6
14	S162P, G422R	7.9
123	S162P, A230E, V323A	9.1
234	A230E, V323A, G422R	10.5
134	S162P, V323A, G422R	10.8
124	S162P, A230E, G422R	10.9
1234	S162P, A230E, V323A, G422R	13.9

Supplementary Tables S10 is part of the Excel workbook

SuppTables_Synechocystis_CbbM-screening.xlsx

Supp. Table S11. First two inflection points of different CbbM variants as determined by nanoDSF. The mean and standard deviation of three technical replicates is given.

CbbM variant code	Amino acid exchanges	Inflection point #1 (°C)	Inflection point #2 (°C)
0	-	44.92 ± 0.04	70.12 ± 0.06
2	A230E	44.17 ± 0.08	69.76 ± 0.03
4	G422R	49.25 ± 0.02	70.00 ± 0.06
14	S162P, G422R	49.72 ± 0.14	69.27 ± 0.26
1234	S162P, A230E, V323A, G422R	52.63 ± 0.14	68.56 ± 0.08

Supp. Table S12. Oligonucleotides used in this study.

Name	Sequence	Function
P01	GGCCACCGGTGTTGTATTGT	Test of integration of complete CRISPRi construct into genome
P02	CTAGCTCACTCGGTCGCTACTAC	Test of integration of complete CRISPRi construct into genome
P03	CCTGTGGTCACGGTTCTGTT	Test of integration of complete CRISPRi construct into genome
P04	GCTTGCAGCACCAACATGAA	Test of integration of complete CRISPRi construct into genome
P05	TGGGATTGTAACAATTTTTAGTG GTCA	Test of full segregation of *ndhD3* deletion strain
P06	CATGGAGGCATAACCCCGTT	Test of full segregation of *ndhD3* deletion strain
P07	TGCCTACCTGAATCAAACTGTCA	Test of full segregation of *ndhD4* deletion strain
P08	TTTCTTTGGCCGTCTCACCA	Test of full segregation of *ndhD4* deletion strain
M01	GGTTCCTGAGGAAATGGTACGC AAG	Site-directed mutagenesis of *Gallionella* Rubisco coding sequence, M140E
M02	AAGAAGTCCAACATGCGTAAAC	Site-directed mutagenesis of *Gallionella* Rubisco coding sequence, M140E
M03	TTTAGGTCGGCCCGAGACGGAC G	Site-directed mutagenesis of *Gallionella* Rubisco coding sequence, S162P
M04	ACTTTCCATAGATTGCTGATATTC	Site-directed mutagenesis of *Gallionella* Rubisco coding sequence, S162P
M05	AGCCCAGCAAGAAACCGGCCAA G	Site-directed mutagenesis of *Gallionella* Rubisco coding sequence, A230E
M06	CGATCCATAGCTTCTGCCAC	Site-directed mutagenesis of *Gallionella* Rubisco coding sequence, A230E
M07	TATGAAGCTCGCCCGCCTAATGG	Site-directed mutagenesis of *Gallionella* Rubisco coding sequence, V323A
M08	TAGCATAGAGGGTCCATG	Site-directed mutagenesis of *Gallionella* Rubisco coding sequence, V323A
M09	TATTAGCCTACGGCAGGCGTATG ACTG	Site-directed mutagenesis of *Gallionella* Rubisco coding sequence, G422R

M10	CCTCCAGCTGCTGGAGAA	Site-directed mutagenesis of *Gallionella* Rubisco coding sequence, G422R
S1	ACTGGAGTTCAGACGTGTGCTCTTCCGATCTTGGCCGCGGTAACTAGTAAGC	PAGE-purified, Illumina library preparation
S2	ACACTCTTTCCCTACACGACGCTCTTCCGATCTNGTCTAGAATCGCCGAAAGTAATTCAACTCC	PAGE-purified, Illumina library preparation
S3	ACACTCTTTCCCTACACGACGCTCTTCCGATCTNNGTCTAGAATCGCCGAAAGTAATTCAACTCC	PAGE-purified, Illumina library preparation
S4	ACACTCTTTCCCTACACGACGCTCTTCCGATCTNNNGTCTAGAATCGCCGAAAGTAATTCAACTCC	PAGE-purified, Illumina library preparation

Supp. Table S13. *Synechocystis* strains used in this study.

Name	Comment	Description
Syn-sgRNA(-)	Wild-type strain transformed with plasmid pMD19T_psbA1_PL22_dCas9_B0015_SpR from (6), which replaces the *slr1181* (*psbA1*) locus by the gene encoding dCas9	Δ*psbA1* P_{J23101}::*tetR* P_{L22}::*SPdcas9*
Syn-sgRNA$_{rbc}$	As *Syn*-sgRNA(-), but with two sgRNAs complementary to the coding sequence of *rbcL*. The sgRNA sequences were obtained from the two strongest acting sgRNAs targeting *rbcL* in (7)	Δ*psbA1* P_{J23101}::*tetR* P_{L22}::*SPdcas9* P_{L22}::*sgRNA_rbcL_1* P_{L22}::*sgRNA_rbcL_2*
Syn-sgRNA$_{rbc}$ *cbbM*(WT)	As *Syn*-sgRNA$_{rbc}$, with RSF1010-based plasmid harboring gene encoding wild-type CbbM, *cbbM*$^{+}$, under control of trc promoter.	P_{trc}::*(N-Strep)-cbbM*$^{+}$ Δ*psbA1* P_{J23101}::*tetR* P_{L22}::*SPdcas9* P_{L22}::*sgRNA_rbcL_1* P_{L22}::*sgRNA_rbcL_2*
Syn-sgRNA$_{rbc}$ *cbbM*(xxxx)	As *Syn*-sgRNA$_{rbc}$ *cbbM*(WT), but with a mutant variant of CbbM encoded instead of wild-type CbbM. xxxx stands for the combination of introduced amino acid exchanges, encoded in the following: 1: S162P, 2: A230E, 3: V323A, 4: G422R	P_{trc}::*(N-Strep)-cbbM(xxxx)* Δ*psbA1* P_{J23101}::*tetR* P_{L22}::*SPdcas9* P_{L22}::*sgRNA_rbcL_1* P_{L22}::*sgRNA_rbcL_2*
cbbM-GFP_{11}|GFP_{1-10}	Wild-type CbbM tagged with 11th alpha helix of split-GFP (8) expressed from trc promoter and encoded on RSF1010-based plasmid, remaining part of GFP (1st to 10th alpha helices (8)) expressed under rhamnose-inducible promoter	Δ*slr0168 cmR* P_{rha}::*spGFP1-10* Ptrc::*(N-Strep)-cbbM-spGFP11*
cbbM-GFP_{11}|noGFP	Wild-type CbbM tagged with 11th alpha helix of split-GFP (8) expressed from trc promoter and encoded on RSF1010-based plasmid, control without remaining part of GFP	Δ*slr0168 cmR* P_{trc}::*(N-Strep)-cbbM-spGFP11*
Syn-sgRNA$_{rbc}$ *cbbM*(WT) Δ*ndhD3* Δ*ndhD4*	As *Syn*-sgRNA$_{rbc}$ *cbbM*(WT), with deletions of genes encoding NDH-1 subunits NdhD3 and NdhD4, encoded by *ndhD3* (*sll1733*) and *ndhD4* (*sll0027*).	P_{trc}::*(N-Strep)-cbbM*$^{+}$ Δ*psbA1* P_{J23101}::*tetR* P_{L22}::*SPdcas9* P_{L22}::*sgRNA_rbcL_1* P_{L22}::*sgRNA_rbcL_2* Δ*ndhD3* Δ*ndhD4*

Supp. Table S14: Molecular weights and extinction coefficients of the Rubisco mutant variants as calculated using Expasy ProtParam.

Mutant variant	Molecular weight [Da]	Extinction coefficient
0	53392.59	61685
4	53491.72	61685
2	53450.62	61685
14	53501.76	61685
1234	53531.74	61685

Supp. Table S15: Spectrophotometric Rubisco assay components, preparation and storage. Storage conditions for ATP, phosphocreatine, NADH, creatine phosphokinase, glyceraldehyde 3-phosphate dehydrogenase and 3-phosphoglyceric phosphokinase were inspired by (9).

Component	Assay concentration	Volume to add	Stock	Water or 1 M EPPS	Storage
$NaHCO_3$ pH 8.4	50 mM	10 µL	500 mM	Water	RT
$MgCl_2$	20 mM	10 µL	200 mM	Water	RT
DTT	0.5 mM	5 µL	10 mM	Water	-20°C
ATP	2 mM	5 µL	40 mM	Water	-80°C
Phosphocreatine	10 mM	5 µL	200 mM	Water	-80°C
NADH	0.5 mM	5 µL	10 mM	EPPS	-80°C
Carbonic anhydrase	0.1 mg/mL	10 µL	1 mg/mL	EPPS	-80°C
Creatine phosphokinase	20 U/mL	10 µL	200 U/mL	EPPS	-80°C
Glyceraldehyde 3-phosphate dehydrogenase	20 U/mL	10 µL	200 U/mL	EPPS	-80°C
3-Phosphoglyceric phosphokinase	20 U/mL	10 µL	200 U/mL	EPPS	-80°C
Ribulose-1,5-bisphosphate	1 mM	10 µL	10 mM	Water	-80°C
RuBisCO	500 nM	10 µL	5 µM	20 mM EPPS +20 mM $MgCl_2$	4°C
Total		100 uL			

Supplemental References

1. Hagemann, M., Song, S., and Brouwer, E.-M. (2021) Inorganic Carbon Assimilation in Cyanobacteria: Mechanisms, Regulation, and Engineering. in *Cyanobacteria Biotechnology*, pp. 1–31, 10.1002/9783527824908.ch1
2. Hackenberg, C., Huege, J., Engelhardt, A., Wittink, F., Laue, M., Matthijs, H. C. P., Kopka, J., Bauwe, H., and Hagemann, M. (2012) Low-carbon acclimation in carboxysome-less and photorespiratory mutants of the cyanobacterium *Synechocystis* sp. strain PCC 6803. *Microbiology*. 158, 398–413
3. Eisenhut, M., von Wobeser, E. A., Jonas, L., Schubert, H., Ibelings, B. W., Bauwe, H., Matthijs, H. C. P., and Hagemann, M. (2007) Long-Term Response toward Inorganic Carbon Limitation in Wild Type and Glycolate Turnover Mutants of the Cyanobacterium *Synechocystis* sp. Strain PCC 6803. *Plant Physiol.* 144, 1946–1959
4. Angermayr S. Andreas, van Alphen Pascal, Hasdemir Dicle, Kramer Gertjan, Iqbal Muzamal, van Grondelle Wilmar, Hoefsloot Huub C., Choi Young Hae, and Hellingwerf Klaas J. (2016) Culturing *Synechocystis* sp. Strain PCC 6803 with N2 and CO2 in a Diel Regime Reveals Multiphase Glycogen Dynamics with Low Maintenance Costs. *Appl. Environ. Microbiol.* 82, 4180–4189
5. Davidi, D., Shamshoum, M., Guo, Z., Bar-On, Y. M., Prywes, N., Oz, A., Jablonska, J., Flamholz, A., Wernick, D. G., Antonovsky, N., de Pins, B., Shachar, L., Hochhauser, D., Peleg, Y., Albeck, S., Sharon, I., Mueller-Cajar, O., and Milo, R. (2020) Highly active rubiscos discovered by systematic interrogation of natural sequence diversity. *EMBO J.* 39, e104081
6. Yao, L., Cengic, I., Anfelt, J., and Hudson, E. P. (2016) Multiple Gene Repression in Cyanobacteria Using CRISPRi. *ACS Synth. Biol.* 5, 207–212
7. Miao, R., Jahn, M., Shabestary, K., Peltier, G., and Hudson, E. P. (2023) CRISPR interference screens reveal growth–robustness tradeoffs in *Synechocystis* sp. PCC 6803 across growth conditions. *Plant Cell*. 10.1093/plcell/koad208
8. Cabantous, S., Terwilliger, T. C., and Waldo, G. S. (2005) Protein tagging and detection with engineered self-assembling fragments of green fluorescent protein. *Nat. Biotechnol.* 23, 102–107
9. Sales, C. R. G., da Silva, A. B., and Carmo-Silva, E. (2020) Measuring Rubisco activity: challenges and opportunities of NADH-linked microtiter plate-based and 14C-based assays. *J. Exp. Bot.* 71, 5302–5312
